# Intelligent Monitoring of Shear Damage Evolution at Bonded Sandstone Interfaces Based on ViT and Piezoelectric Ultrasonic Testing

**DOI:** 10.3390/s26175586

**Published:** 2026-09-02

**Authors:** Jiancheng Liu, Chong Wang, Hongbo Zhang, Zhongshan Zhang, Dong Xu, Hongyu Zou, Zhenbin Xie

**Affiliations:** 1 School of History and Culture, Sichuan University, Chengdu 610064, China; liujc90@163.com (J.L.); xd1212xd@163.com (D.X.); 2 Sichuan Provincial Institute of Cultural Relics and Archaeology, Chengdu 610041, China; wangchonglican@126.com (C.W.); hirostar@163.com (H.Z.); zzssxsz@163.com (Z.Z.); zouhy18@163.com (H.Z.); 3 Sichuan Museum, Chengdu 610000, China

**Keywords:** bonded sandstone interfaces, damage intelligent monitoring, piezoelectric ultrasonic testing, vision transformer

## Abstract

**Highlights:**

**What are the main findings?**

- Continuous Wavelet Transform (CWT) spectra and wavelet packet energy changes tracked shear damage evolution at bonded sandstone interfaces.
- A lightweight CWT-Vision Transformer (CWT-ViT) with Stage I healthy-reference alignment was proposed for small-sample cross-specimen identification of damage stages.
- Robust identification of damage stages across specimens was achieved under fixed specimen-wise evaluation.

**What are the implications of the main findings?**

- It enables dynamic monitoring and optimal intervention timing for tunnel interfaces.
- It informs targeted protection and early warning for similar interfaces in tunnels.

**Abstract:**

This paper presents a method for monitoring damage evolution at sandstone-binding material interfaces by combining a Vision Transformer (ViT) deep learning model with piezoelectric ultrasonic monitoring. Direct shear tests were conducted on bonded weak sandstone specimens. The results indicate that the damage evolution process can be divided into four stages: initial elastic, compaction and stabilization, crack propagation and coalescence, and frictional sliding and interlocking. Ultrasonic signals acquired during loading reveal that interface damage evolution is in good agreement with the time-domain waveforms, Continuous Wavelet Transform (CWT) time–frequency spectra, and wavelet packet energy. Based on the ViT-Small/16 backbone, a ViT model with adaptive frequency-feature extraction was developed for small-sample and cross-specimen interfacial damage-stage identification, using the Stage I health observations of each specimen prior to loading as the reference. Results from five repeated runs with different random seeds show that the method achieved an accuracy of 93.23% ± 0.72% and an F1-score of 92.32% ± 0.90%, demonstrating favorable recognition performance and stability under the current condition. This study offers insights into the damage monitoring and subsequent warning of similar binary interfaces in tunnel engineering, geotechnical engineering, and stone cultural heritage conservation.

## 1. Introduction

**Figure 1:**
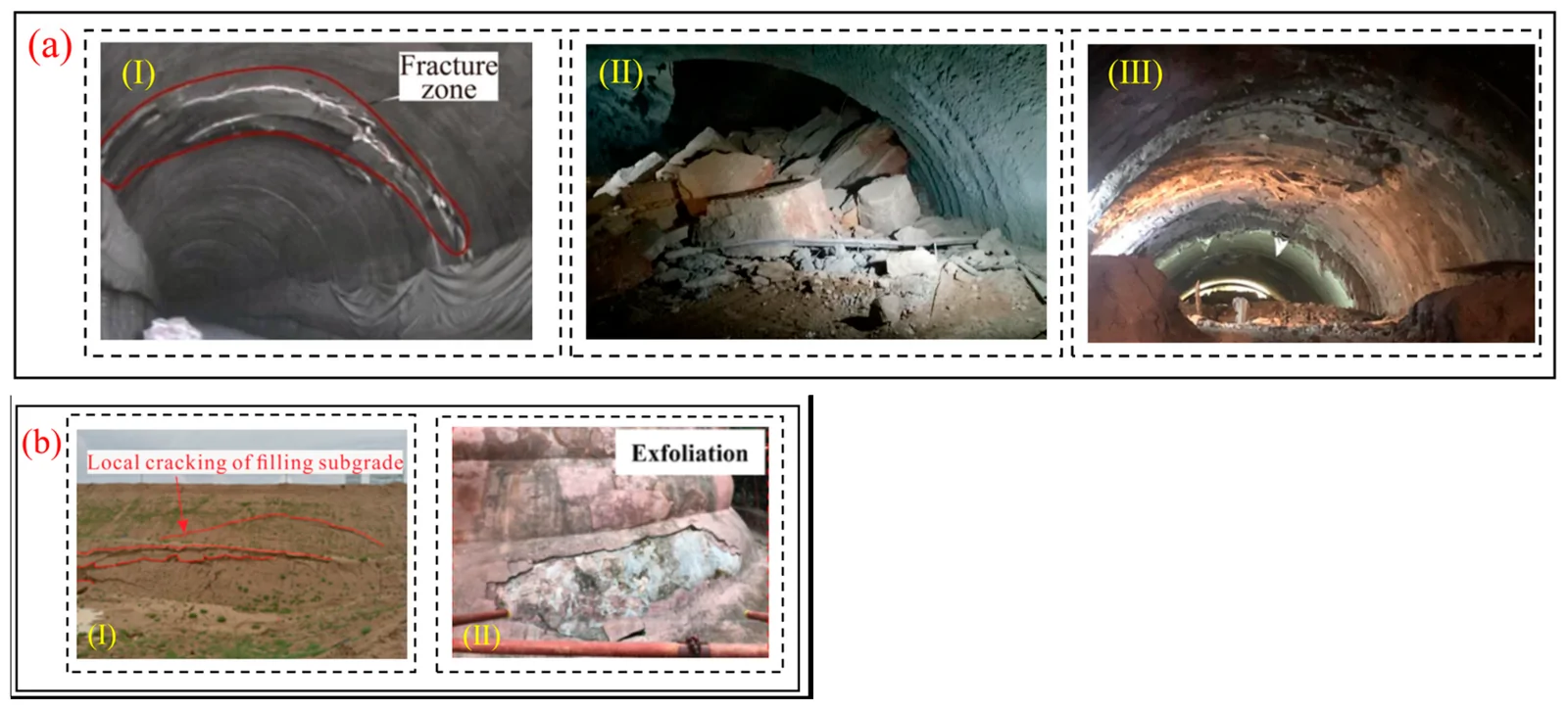
Examples of damage and failure at binary interfaces: (**a**) Tunnel Engineering: (**I**) concrete interface damage. Reprinted from [1] (CC BY 4.0), and from [2], Copyright 2014, with permission from Elsevier. (**II**) Collapse of a tunnel roof. Reprinted from [3], Copyright 2023, with permission from Elsevier. (**III**) Collapse of a tunnel roof (T341 tunnel, Turkey). Reprinted from [4], CC BY 3.0. (**b**) Other Engineering Industries: (**I**) Damage to Loess and Bedrock Slopes. Reprinted from [5]. (**II**) Detachment of a stone cultural relic from its restoration layer. Reprinted from [6], Copyright 2022, with permission from Elsevier.

In engineering practice, when two materials with distinct engineering properties come into contact and work together, they form a typical binary composite structure. Such binary interfaces are widespread in tunnel engineering, for example, between soft and hard rock layers, between rock bolts and the surrounding rock, between the excavated rock mass and the primary support, and between the primary support and the secondary lining. Typical examples include rock–concrete, rock–soil, and rock–binding material interfaces. Owing to the discontinuity and abrupt change in mechanical properties across the interface, this interfacial region often acts as a primary source of stress concentration and damage initiation, representing a critical weak link that limits the overall load-bearing capacity and long-term durability of the structure. In actual service environments, such interfaces not only endure long-term synergistic degradation driven by multiple coupled factors—such as loads, freeze–thaw cycles, and alternating wet–dry conditions—but may also be subjected to extreme events such as earthquakes, high hydraulic gradients, and landslide unloading. This can easily induce macroscopic damage such as interface displacement, slippage, and even delamination, thereby jeopardizing the overall safety of tunnel structures (Figure 1a). Furthermore, similar interface failure issues also exist in geotechnical engineering and stone cultural heritage conservation (e.g., at the interface between the substrate of stone cultural relics and the restoration layer) (Figure 1b). Therefore, a thorough understanding of the damage evolution of binary interfaces in tunnel engineering under complex environmental conditions, coupled with real-time monitoring and accurate assessment of early-stage damage, is essential not only for dynamic structural health monitoring and determining the optimal timing of intervention but also for providing a critical scientific basis to inform targeted protective strategies. This understanding also offers valuable insights into the monitoring and early warning of similar interfaces in geotechnical engineering and stone cultural heritage conservation.

In recent decades, with the rapid development of high and new technologies, various nondestructive testing methods have been applied in the field of damage monitoring [7,8,9,10,11,12]. Currently, research on nondestructive monitoring technologies for interfacial damage and delamination primarily encompasses microscopic characterization techniques, acoustic emission (AE) technology, and piezoelectric ceramic sensor technology. Microscopic characterization techniques mainly rely on methods such as computed tomography (CT), nuclear magnetic resonance (NMR), and scanning electron microscopy (SEM) to analyze the internal microstructure during the damage evolution process. For example, Shen et al. [13] used nuclear magnetic resonance (NMR) to study the microscale delamination process at the sandstone–concrete interface under freeze–thaw cycles; Wang et al. [14] investigated microstructural changes at the interface under varying degrees of freeze–thaw damage through CT scanning and SEM analysis; Wang et al. [15] simulated the damage evolution and crack propagation at the ultra-high-performance concrete–ordinary concrete interface by combining CT scanning with finite element analysis; Wang et al. [16] analyzed the microstructural damage patterns and evolution processes at the bonding interface of solid rocket engines using in situ micro-CT. Although these methods have effectively revealed the evolution of interfacial damage, the equipment is expensive, the operating conditions are demanding, and the studies are typically limited to laboratory environments, making in situ monitoring difficult to conduct.

Acoustic emission (AE) technology captures the elastic waves emitted by a material during loading-induced deformation or fracture, from which damage information is extracted to analyze crack initiation and propagation [17,18]. Wang et al. [12] and Hu et al. [19] used AE technology to analyze the shear damage evolution at the reinforced concrete-sandstone interface; Shen et al. [20] used acoustic emission technology to analyze the damage evolution at the interface of concrete-encased CFST columns; Wang et al. [21] employed fiber Bragg grating (FBG) sensors and acoustic emission technology to elucidate the failure characteristics at the interface of anchored structural specimens. Although AE technology can effectively capture internal damage, its signal characteristics are strongly influenced by material properties, data interpretation relies heavily on extensive databases and experience, and it suffers from poor noise immunity and relatively high cost. In interface damage monitoring, AE technology still faces limitations: damage-sensitive features are easily masked by noise; it struggles to accommodate the non-stationary nature of damage evolution, and it lacks physical constraints in the feature space [9]. Therefore, it often needs to be combined with other NDT methods.

In contrast, active sensing technology based on lead zirconate titanate (PZT) piezoelectric ceramics offers advantages such as rapid real-time response, low cost, high sensitivity, high adaptability, and broad applicability [22,23,24], and it has been widely applied in grouting quality monitoring of concrete [25,26], structural health monitoring [27,28], corrosion and degradation monitoring of reinforced concrete structures [29,30], and concrete strength development monitoring [31,32,33]. The core principle relies on the piezoelectric effect of PZT: PZT patches act as both ultrasonic transmitters and receivers, and damage is identified, localized, and assessed by analyzing changes in the transmitted ultrasonic signals [34]. In recent years, many researchers have adopted this approach for interface damage monitoring. Wu et al. [35] identified delamination between concrete and reinforcing bars using embedded piezoelectric ceramic sensors and actuators. Yang et al. [36] employed wavelet packet decomposition, power spectral density, and short-time Fourier transform to compare monitored signals at different curing ages, establishing a mapping relationship between wavelet packet energy and bond strength, wherein they directly determined the bond strength at the carbon-fiber-reinforced polymer (CFRP)–wood column interface. Liu et al. [37] embedded PZT transducers in aggregates to transmit and receive stress waves and used wavelet packet analysis to monitor delamination at the CFRP–concrete interface. Xu et al. [38] combined wavelet packet analysis with piezoelectric sensing to achieve active detection of delamination at the steel pipe-concrete interface. Qian et al. [39,40] quantitatively assessed the extent of delamination damage at the interface of composite concrete beams based on damage indices extracted from wavelet packet decomposition. Simultaneously, they used piezoelectric smart aggregates to actively monitor the separation process between the steel plate and the confinement plate in a novel steel-plate shear wall. Chen et al. [41] proposed a detection method that combines PZT surface wave measurements with numerical simulation to identify delamination damage at the interface of concrete-filled steel tube members. Zhao et al. [42] employed PZT-based ultrasonic monitoring to continuously track the evolution of slip damage at the steel-ultra-high-performance concrete (UHPC) interface during static push-off tests. They found a strong correlation between the damage index extracted via wavelet packet analysis and the progression of interfacial slip. Jiang et al. [43] used PZT-based active sensing, combined with fast Fourier transform and wavelet packet energy analysis, to successfully identify the damage state at the interface of steel-concrete composite structures. The above studies have clearly demonstrated the advantages and feasibility of piezoelectric ceramic-based ultrasonic testing for interfacial damage monitoring. However, this technology is not yet fully mature, and its applicability to various types of interfacial damage remains to be validated. For example, to date, few studies have reported on the monitoring of damage and delamination at rock–binding material interfaces (e.g., grout-rock interfaces in tunnel engineering and interfaces between the substrate of stone cultural relics and restoration materials, etc.).

In nondestructive testing, Fourier transforms and wavelet packet analysis are commonly used for feature extraction. These methods can describe signal variations in the frequency domain or time–frequency domain, but they typically rely on predefined analysis parameters and manual feature selection; as a result, the analysis results may be influenced by parameter settings and subjective judgment [44,45]. Machine learning algorithms, particularly deep learning methods, offer new approaches to overcoming this limitation. For example, Hu et al. [46] utilized Fuzzy C-Means (FCM) clustering combined with Random Forest (RF) to classify acoustic emission events in steel–UHPC composite structures, achieving a recognition accuracy of 92.9% for three damage mechanisms; Guo et al. [47] employed self-organizing maps to cluster the RA-AF dataset, thereby more effectively distinguishing between two types of damage cracks at the rock–concrete interface. Although such methods have improved identification efficiency, their inputs still rely primarily on manually constructed feature parameters. To reduce the need for manual feature design, some studies have directly used one-dimensional deep neural network models to learn damage characteristics from one-dimensional responses. Zhang et al. [44] used NiVGG-Net to automatically recognize acoustic emission waveforms, achieving classification of damage in ultra-high-performance fiber-reinforced cementitious composites and interfacial damage with an accuracy, exceeding 90%. Deng et al. [48] used 1D-CNN and BiLSTM models to achieve an accuracy of 97.6% in identifying damage in CFRP-reinforced concrete structures. Hacıefendioğlu et al. [49] utilized one-dimensional vibration responses obtained from visual measurements. They combined time-domain and frequency-domain information using the Fast Fourier Transform (FFT) and integrated one-dimensional convolutional encoding with Variational Autoencoders (VAE) to achieve unsupervised damage identification in steel frame structures. These studies show that deep learning methods leveraging one-dimensional responses can effectively learn damage-related features, which is crucial for identifying structural damage. While these studies have partially incorporated frequency-domain information, their characterization primarily focuses on one-dimensional responses, lacking a comprehensive representation of local time–frequency evolution during interfacial damage processes. In interfacial damage scenarios, the ultrasonic response not only shows changes in waveform amplitude and localized time-series structure but also reflects the evolution of frequency content and energy distribution. Therefore, to simultaneously preserve both temporal localization and frequency distribution information, researchers often convert one-dimensional ultrasonic signals into two-dimensional time-frequency representations. Compared to directly modeling raw one-dimensional waveforms, this representation facilitates the extraction of discriminative features related to damage evolution by visual models. In damage detection studies based on two-dimensional representations, Zhang et al. [50] processed time-series signals into two-dimensional time-frequency spectra and combined them with a CNN to achieve delamination monitoring at the concrete–rock interface with an accuracy exceeding 98%; however, their model training was primarily based on a single specimen. Huang et al. [51] processed the modal vibration modes of composite laminates into two-dimensional Continuous Wavelet Transform (CWT) spectra and used a CNN for delamination detection, achieving an accuracy of 93.89%. Their training samples were primarily derived from finite element simulations, where initial signal variations were relatively controllable. Although existing two-dimensional characterization studies have validated the feasibility of these methods, their data sources are often derived from single or relatively controlled conditions, and the applicability of these methods to identification across different specimens remains to be further investigated. With the development of Vision Transformers (ViT) and its variants, they have gradually been applied to structural health monitoring driven by two-dimensional sensor images. Hiçyılmaz [52] utilized CWT spectra generated from a 72-member steel truss model and measurements from a real steel truss bridge. By combining these with an adaptive Bayesian optimization process to optimize the ViT architecture, an accuracy of 98.43% was achieved for monitoring steel truss structures. Xin et al. [53] converted the raw signal into CWT time–frequency spectra and used the Swin Transformer to achieve high-precision structural damage identification; Du et al. [54] used a piezoelectric sensor array to excite and receive guided wave (GW) signals in curved composite laminates, converting one-dimensional signals into two-dimensional images, which were then fed into an improved Convolutional Vision Transformer (CvT) for damage localization; this method achieved a localization accuracy of 98.1%. Hu et al. [55] converted Lamb wave signals into CWT time–frequency spectra as input and utilized a CNN-ViT model to automate damage identification in carbon-fiber-reinforced polymers, demonstrating both accuracy and reliability. This model has also been applied to research on interfacial damage. Li et al. [56] converted AE signals into two-dimensional image data via the Gramian angular field and used a ViT with an improved adaptive feature fusion module to achieve high-precision damage identification in CFRP-PMI laminate structures.

The above studies indicate that converting one-dimensional sensor signals into two-dimensional time-frequency or image representations and training them using visual models is a viable approach for effectively identifying complex damage features. However, current research on CWT-ViT has primarily focused on damage detection or localization in structures such as steel trusses and composite laminates. In contrast, studies on the identification of continuous damage evolution at the bonded weak sandstone-binding material interface under direct shear loading remain scarce. Compared to relatively regular and homogeneous structures, this type of interface system is simultaneously influenced by the heterogeneity of sandstone, variations in surface roughness, and the randomness of crack propagation paths. Consequently, its piezoelectric ultrasonic response exhibits complex time–frequency coupled evolution characteristics, and there are continuous transitions between different damage stages. Meanwhile, due to limitations in specimen preparation, direct shear testing, and ultrasonic data acquisition conditions, relevant studies often struggle to obtain large-scale specimen data with a balanced distribution across damage stages; differences in initial conditions, sensor coupling, and signal energy scales among specimens further increase the difficulty of cross-specimen identification.

This study aims to address the aforementioned research gap by investigating the applicability of combining piezoelectric ultrasonic monitoring with the ViT for detecting damage evolution at the weak sandstone-binding material bonded interface. Direct shear tests were conducted using a custom-designed shear test fixture on bonded interfaces formed between weak red sandstone specimens with varying surface roughness and binding materials, and the shear behavior and damage evolution of the interfaces were systematically analyzed. During shearing, PZT patches bonded to both sides of the interface served as an actuator and a receiver, respectively, to transmit and receive stress wave signals. Furthermore, the signal attenuation characteristics during shear damage were systematically analyzed by integrating the time-domain waveforms, CWT time–frequency spectra, and wavelet packet energy. Finally, using the Stage I health observations of each specimen as a reference, a ViT model with adaptive frequency-feature extraction was developed for small-sample and cross-specimen interfacial damage-stage identification, thereby enabling intelligent identification and monitoring of the interface damage evolution process. This study provides insights into the damage monitoring of similar binary interfaces in tunnel engineering, geotechnical engineering, and the conservation of stone cultural heritage. It lays the groundwork for future research on online early-warning methods.

## 2. Experiment Methodology

### 2.1. Fundamental Principles of Piezoelectric Ultrasonic Monitoring

**Figure 2:**
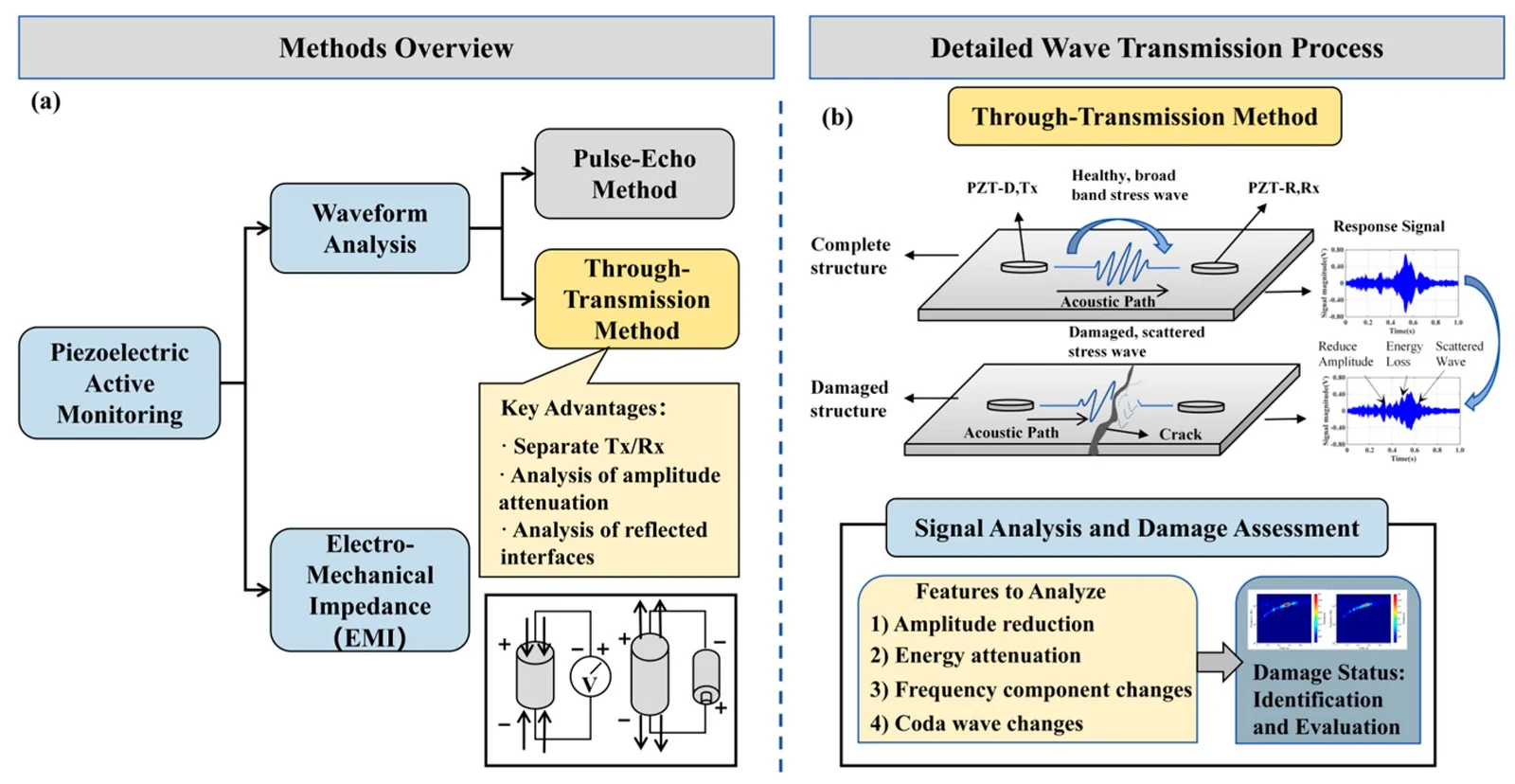
Schematic illustration of the piezoelectric-based wave through-transmission active monitoring method: (**a**) classification of methods; (**b**) Detailed Wave Transmission Process.

Active structural health monitoring (SHM) methods based on piezoelectric ceramics are primarily divided into two categories: the wave method and the mechanical impedance method. This study adopts the wave method for damage monitoring. The basic principle is as follows: two piezoelectric ceramic transducers are used as a driver and a sensor, respectively. The driver utilizes the inverse piezoelectric effect to excite specific stress wave modes within the structure. When the stress wave encounters damage during propagation, its characteristic parameters, such as propagation time, amplitude, mode, or energy, will change. The sensor then uses the direct piezoelectric effect to convert the stress wave carrying damage information into an electrical signal. By comparing the response signal with the original excitation signal, the internal damage state of the structure can be identified and assessed. Piezoelectric wave analysis methods primarily include the pulse-echo method and the transmission method. This study uses the transmission method (i.e., a single-transmitter, single-receiver operating mode) to determine the presence of internal defects in a structure by analyzing the amplitude attenuation or energy loss of the received signal. The physical basis for this method is that damage causes reflection, scattering, and diffraction of stress waves, thereby significantly weakening the transmitted signal intensity, see Figure 2.

Commonly used time–frequency analysis methods for ultrasonic signal feature extraction include the Fourier transform, wavelet transform, and wavelet packet transform. Among these, the CWT produces a time–frequency spectrum that captures the time-varying frequency content of a signal by continuously scaling and shifting the mother wavelet. It is particularly well suited for analyzing localized transient and non-stationary components caused by damage in ultrasonic signals [57]. Therefore, this paper employs the CWT for time–frequency analysis. Its mathematical definition is given in Equation (1).

(1) $$\mathrm{CWT}(a,b)=\int_{-\infty }^{\infty }\frac{1}{\sqrt{\left|a\right|}}x(t)\bar{\psi }(\frac{t-b}{a})$$

where x(t) represents the original time-domain signal function; a and b denote the scaling and translation parameters, respectively; ψ(t) is the mother wavelet function; and $\bar{\psi }(t)$ is the complex conjugate of ψ(t). Commonly used wavelet functions include Haar, Daubechies, Mexican hat, Morlet, and Meyer. The Morlet wavelet is adopted in this paper because of its strong time–frequency localization capability, high adaptability, and its widespread use in CWT analysis.

This paper adopts wavelet packet decomposition to quantitatively analyze the response signals by extracting wavelet packet energy over different time intervals, aiming to reveal the energy evolution patterns during interface damage. The specific procedure is as follows: The original signal S was decomposed by an n-level wavelet packet decomposition using the db4 wavelet, yielding a total of 2*^n^* reconstructed signals (*S*_1_, *S*_2_, *S*_3_, …, *S*_2_*^n^*) after *n* levels. The reconstructed signal at the *i*-th frequency band and the *j*-th time step of the *n*-th level is denoted as *S_i,j_*. If *m* (1 *≤ m ≤* 2*^n^*) is defined as the number of sampling points of the signal, then *S_i,j_* can be expressed as:

(2) $$S_{i,\;j}=\left(X_{i,\;j,1},X_{i,\;j,2},X_{i,j,\;3},...,X_{i,\;j,m}\right)$$

The energy of the signal samples at the *i*-th frequency band of the *n*-th level is given by:

(3) $$E_{i,j}={\left\|S_{i,\;j}\right\|}^{2}=\sum_{k=1}^{m}X_{i,j,k}^{2}$$

The total wavelet packet energy of the signal at time *j* is given by:

(4) $$E_{j}=\sum_{i=1}^{2^{n}}E_{i,j}=E_{1,j}+E_{2,j}+E_{3,j}+\ldots +E_{2^{n},j}$$

In general, CWT produces time–frequency spectra in which the color represents the magnitude of the wavelet coefficients. In contrast, wavelet packet decomposition serves for the quantitative analysis of frequency-band energy.

### 2.2. Test Material and Sample Preparation

**Figure 3:**
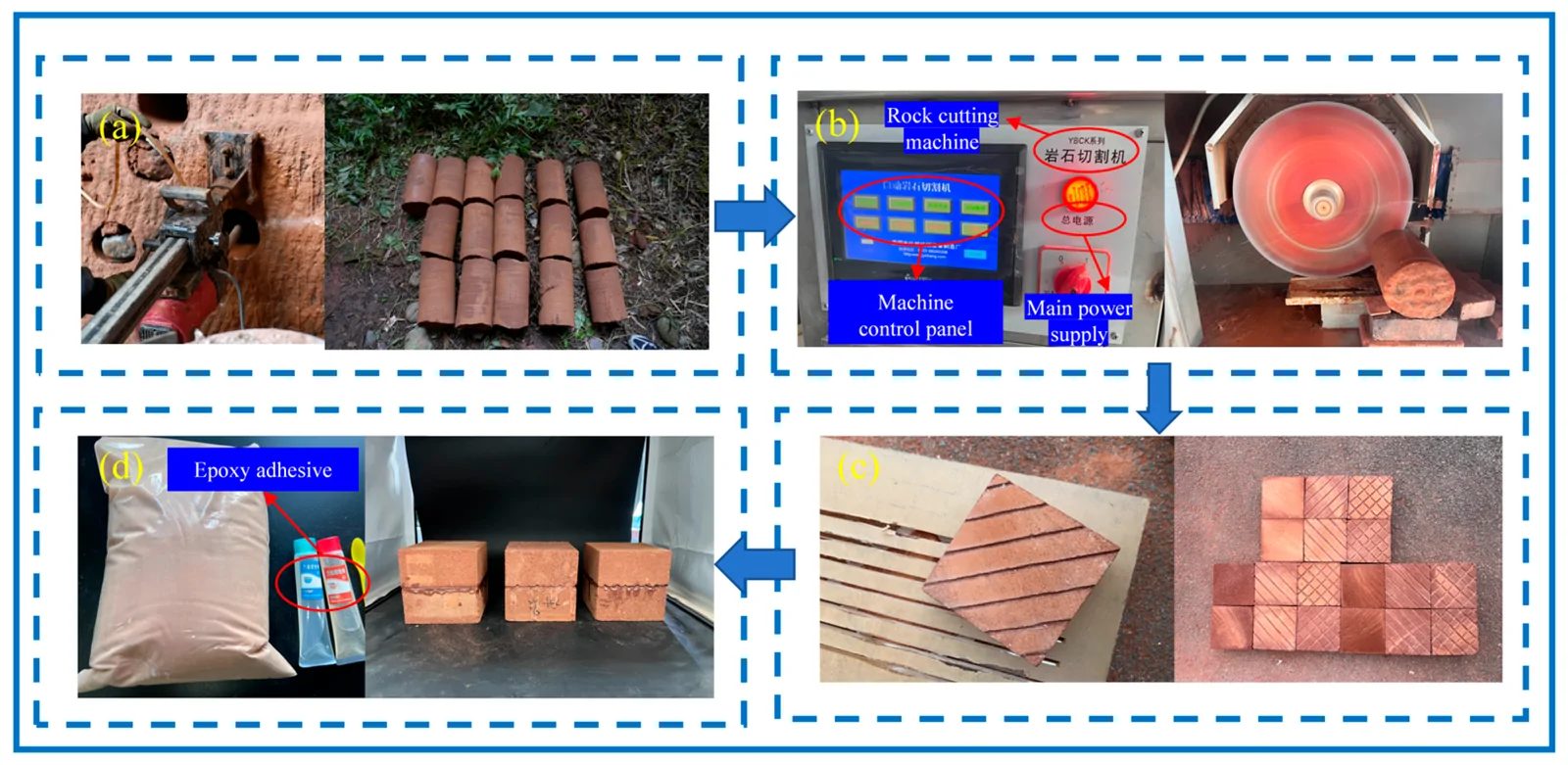
Sample preparation flowchart: (**a**) on-site core sampling; (**b**) sample cutting; (**c**) cutting notches; (**d**) specimen bonding.

In this study, red sandstone sourced from the Leshan area was used as the matrix material, and an epoxy resin adhesive served as the bonding agent. Regarding the bonding mechanism of the epoxy resin, Wen et al. [58] demonstrated through SEM and EDS analyses that the epoxy adhesive can penetrate the surface pores and microcracks of cement-based materials, forming a continuous and dense interfacial transition zone after curing, which enhances mechanical interlocking and stress transfer at the interface. Given that both sandstone and cement mortar are porous materials, the bonding mechanism revealed in that study also provides valuable insight into the epoxy resin-sandstone interface. Due to the length constraints of this paper, however, a deeper discussion of the interfacial interactions will not be expanded upon here and will be explored in future work. The test specimens were 100 mm cubes, each consisting of two red sandstone blocks with cross-sectional dimensions of 100 mm × 100 mm and a thickness of 50 mm, bonded together with the epoxy resin adhesive. To simulate interfaces with different roughness levels, a mechanical grooving method was employed to create regular grooves on the bonding surfaces. The grooves had a width and depth of 2 mm and a fixed spacing of 10 mm. Two grooving patterns were employed: unidirectional grooves and bidirectional grooves. Accordingly, three types of interfacial roughness were defined, in ascending order of roughness: the as-cut surface, the unidirectional-grooved surface, and the bidirectional-grooved surface. Two specimens were prepared for each type, yielding a total of six. The specimen preparation process was as follows: First, red sandstone cores were drilled on-site and then cut to the specified dimensions. Next, the bonding surfaces were grooved using a custom-designed grooving mold and equipment according to the preset parameters. Finally, the blocks were bonded together with the epoxy resin adhesive mixed in the specified ratio and cured under ambient conditions for 7 days before testing, see Figure 3.

### 2.3. Loading Modes and Experiment Procedures

The tests were conducted using the large-scale dynamic performance testing machine for multi-joint rock mass structures at Chengdu University of Technology, a custom-made interface shear fixture, and ultrasonic monitoring equipment. The testing machine consists of a hydraulic power unit, a vertical loading device, and a computer-controlled data acquisition system. It has a normal displacement stroke exceeding 100 mm and a displacement loading rate ranging from 0.005 mm/min to 100 mm/s. Its primary function is to provide a stable vertical shear load for the test. The custom-made interface shear fixture comprises a small FPY-101 hydraulic press (Yuhuan Changyou Hydraulic Tools Co., Ltd., Taizhou, China), an HZC-H1 pressure sensor (Anhui Chengying Sensor Co., Ltd., Bengbu, China), lateral support plates, a base plate, tie rods, and an upper load-bearing plate, with a maximum load capacity of approximately 100 kN. Its primary function is to provide direct shear conditions for specimens. The ultrasonic monitoring system consists of PZT piezoelectric ceramic patches, a signal exciter, a charge amplifier, a data acquisition unit, and a monitoring terminal. Its primary function is to perform ultrasonic monitoring of the specimens during loading. The PZT patches were commercially available circular wafers with a diameter of 10 mm, a thickness of 1 mm, and a density of 7.6 × 10^3^ kg/m^3^. Their piezoelectric constants *d*_31_ and *d*_33_ were 3.15 × 10^−10^ C/N and 2.96 × 10^−10^ C/N, respectively, and their dielectric permittivity was 2.77 × 10^−8^ F/m.

**Figure 4:**
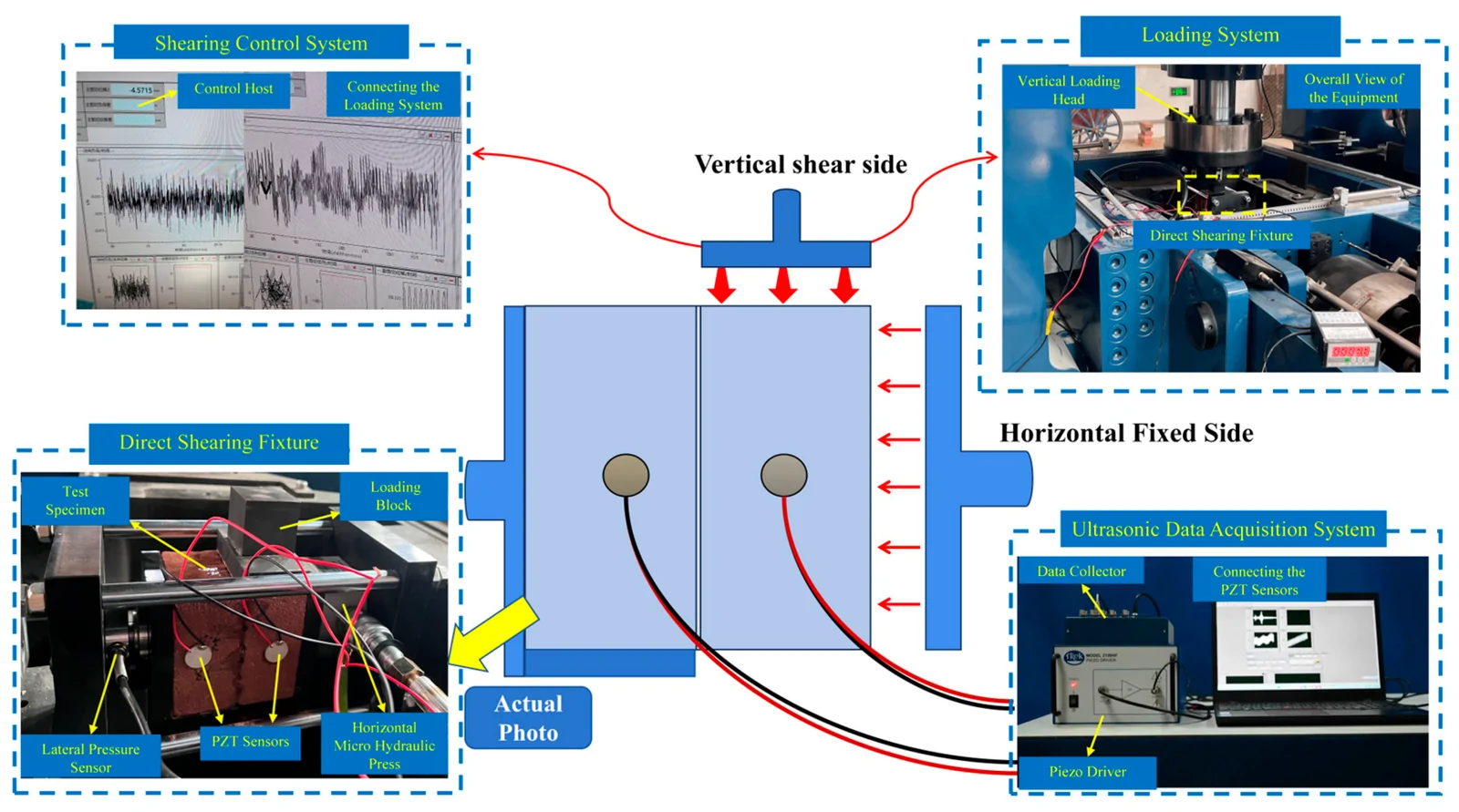
Test equipment schematic diagram.

The specific test procedure was as follows: First, the PZT patches were pretreated by soldering wires to their electrodes and connecting them to the ultrasonic monitoring system interface. Next, two PZT patches were used as the actuator and sensor, respectively, and bonded to predetermined positions on the test specimen using epoxy resin. The two PZT patches were arranged symmetrically on either side of the bonded layer, with a fixed center-to-center distance of 60.0 mm, to maintain a consistent path length under the current test conditions and to obtain stable and relatively clear ultrasonic responses related to the interface. Finally, the specimen was placed in a computer-controlled servo rock shear tester. In accordance with GB/T 50266-2013 [59] and relevant research experience [19,60,61], a transverse load of 0.01 MPa was first slowly applied and held constant to stabilize the specimen and prevent issues such as tilting during shearing; then, a shear load was applied at a rate of 0.5 mm/min until the specimen failed. During shearing, the actuator emitted a sinusoidal swept-frequency signal with a sweep range of 1–400 kHz and a peak amplitude of 10 V. Based on the typical frequency characteristics of similar disc-shaped PZT patches, the radial resonant frequency is approximately 200 kHz; therefore, the sweep range in this study was set to 1–400 kHz to encompass the frequency band near the expected radial resonance. The signal sampling frequency was set to 1 MHz, with a sampling duration of 1 s; the corresponding theoretical Nyquist frequency is 500 kHz, which is higher than the upper limit of the swept-frequency excitation. Additionally, to obtain the strength parameters of the primary red sandstone, rapid shear tests were conducted on primary red sandstone specimens without bonded interfaces using a conventional computerized servo rock testing machine, yielding a measured peak strength of 3.64 MPa, see Figure 4.

## 3. Damage Evolution and Ultrasonic Signal Processing Analysis

### 3.1. Mechanical Evolution Characteristics of Interface Shear

**Figure 5:**
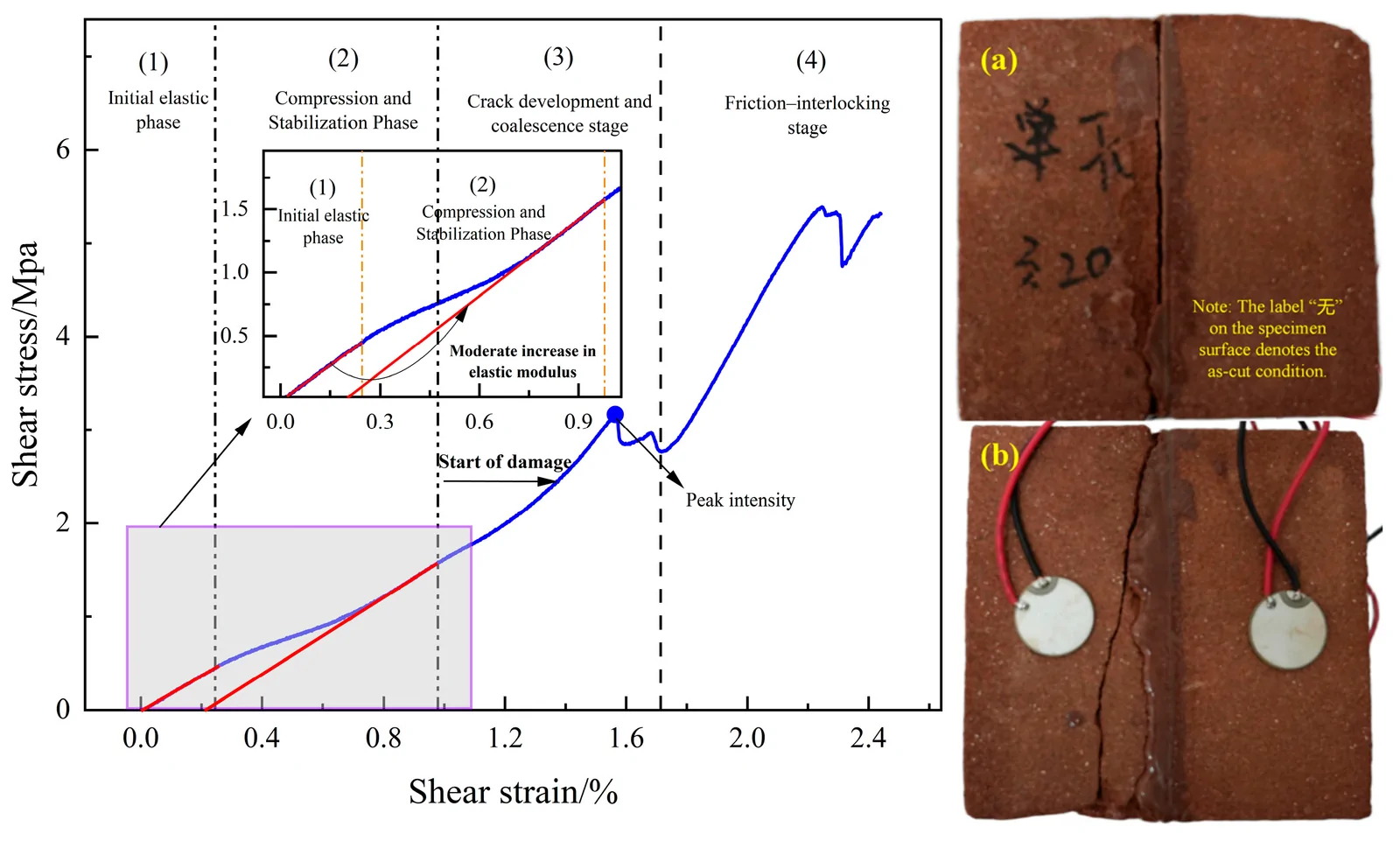
As-cut specimen stress–strain curve: (**a**) top view; (**b**) side view.

**Figure 6:**
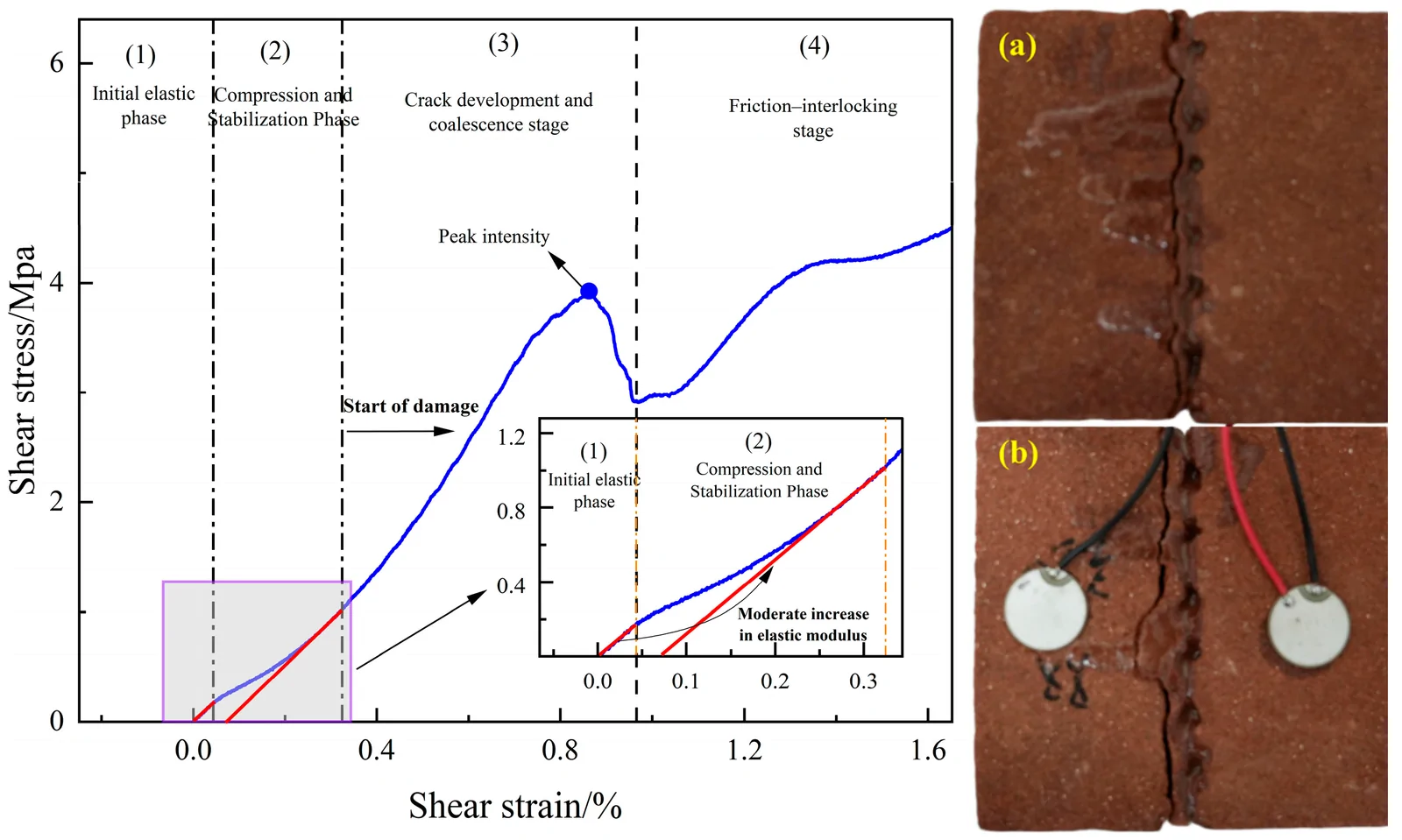
Bidirectional-grooved groove specimen stress–strain curve: (**a**) top view; (**b**) side view.

Figure 5 and Figure 6 present the stress-strain curves for the as-cut specimen and the bidirectional-grooved specimen, respectively. As shown in Figure 5, the stress–strain curve of the as-cut specimen exhibited strain softening characteristics and two peaks, with the first peak reaching a strength of 3.15 MPa. Based on the stress–strain curve characteristics and real-time observations, the damage evolution of the specimen during shearing was divided into four stages. Stage I was the initial elastic stage: when the stress was below approximately 0.49 MPa, the slope of the curve remained essentially constant, and only elastic deformation occurred within the specimen. Stage II was the compaction and stabilization stage: as strain increased, the slope of the curve first decreased slightly and then stabilized. The slope during this stage was slightly greater than that in Stage I, indicating that microscopic cracks and pores within the specimen were gradually compacted, increasing structural stiffness. Stage III was the crack propagation and coalescence stage: as strain continued to increase, the slope of the curve increased accordingly. New cracks were observed to initiate during the test. After the stress reached its first peak, the cracks coalesced rapidly, and the curve immediately entered the strain-softening stage. Stage IV was the frictional sliding and interlocking stage: as strain increased further, the already separated, rough fracture surfaces continued to bear load through frictional sliding and interlocking, and the main cracks propagated further along the vertical shear plane until a macroscopic through-going crack was formed. The final shear failure of the specimen occurred within the sandstone matrix, rather than at the bonded interface. At failure, two dominant cracks formed: one was an oblique crack propagating away from the interface, and the other was a vertical crack developing near the interface; these two cracks intersected in the middle.

As shown in Figure 6, the stress–strain curve of the bidirectional-grooved specimen was similar in overall characteristics to that of the as-cut specimen, also exhibiting two peaks, with the first peak strength of 3.91 MPa; the damage evolution process could also be divided into four stages. However, three differences were observed in the details. Specifically: the initial modulus in stage I differed; in Stage III, the modulus of the bidirectional-grooved specimen first increased and then decreased until reaching the first peak; and the first peak strength of the bidirectional-grooved specimen was slightly higher than that of the as-cut specimen. The ultimate failure of the specimen also occurred within the sandstone matrix. The dominant crack was a vertical crack that developed near the interface, propagating from both ends toward the center and away from the interface.

Based on the macroscopic failure location, the failure observed in the current tests presents primarily as near-interface shear failure controlled by the sandstone matrix, rather than macroscopic debonding at the epoxy-sandstone interface. Under shear loading, the load is transmitted through the bonded region to the near-interface sandstone. Owing to local heterogeneity caused by sandstone pores, natural microcracks, and grain contacts, certain local regions may reach their shear strength limit first, thereby inducing crack initiation and propagation within the sandstone matrix.

The above analysis indicates that the stress–strain curves of the as-cut and bidirectional-grooved specimens exhibit similar behavioral characteristics, revealing a clear pattern. The first peak strength of both specimen types is close to the shear strength of the intact sandstone; thus, it can be inferred that in interface shear tests on bonded specimens, the first peak serves as an important marker of the rapid coalescence of the main crack and the onset of the post-peak damage stage. However, these stress–strain curve characteristics differ to some extent from those reported in other interfacial shear or triaxial shear tests. The main differences are as follows: the curves exhibit two distinct peaks; and during the early damage stage, the modulus either increases monotonically or first increases and then decreases. Similar phenomena have also been reported in previous studies. For example, two peaks and the phenomenon of modulus rebound or remaining constant after the first peak were present in the interface shear tests of Liu et al. [61] at the shear zone-bedrock step interface. Their study provided a preliminary analysis, but a thorough analysis was not conducted. Similarly, two peaks were present in the oblique shear tests of Shen et al. [62] at the concrete–rock interface, but a detailed analysis was not provided. In light of the actual conditions of this experiment, the above phenomenon may be tentatively explained as follows: the lateral side of the shear fixture acts as a fixed boundary. Once a crack forms, as the vertical shear load continues to increase, the lateral confinement effect may gradually strengthen, causing the lateral confining pressure to increase accordingly. Under these conditions, the contact, friction, and mechanical interlocking between the rough fracture surfaces and the micro-protrusions may intensify, increasing apparent stiffness and, to some extent, suppressing the shear dilation effect, causing the slope of the curve to rise rather than fall. Simultaneously, the crack propagation path may also change with the constraint state, gradually shifting from propagation near the interface to oblique propagation. When the stress reaches the first peak, localized coalescence may occur; thereafter, the frictional effects of the rough fracture surfaces, the mechanical interlocking of the micro-protrusions, and the residual bonding in the non-coalesced regions may still provide a certain load-bearing capacity, causing the stress to continue increasing with strain and ultimately forming a second peak.

In addition, certain differences were observed in the stress-strain curves and failure modes between the two specimen types. This was primarily because the bonded interface possessed sufficiently high strength, so that all failures occurred within the sandstone matrix. Consequently, the actual strength was governed by the sandstone itself, and the influence of interfacial roughness was relatively minor. The red sandstone used in this experiment was obtained from the Jiaguan Formation of the Upper Cretaceous System. This sandstone is characterized by low strength, high porosity, weak cementation, and significant anisotropy. Although samples were selected from a moderately weathered zone and care was taken to ensure that both specimens were taken from the same core and oriented in the same direction, the test results indicated that the mechanical properties of the sandstone remained difficult to control effectively. In terms of failure modes, the as-cut specimens exhibited an interlaced pattern of oblique and vertical cracks.

The oblique cracks coalesced first, leading to the first peak. Subsequently, the vertical cracks gradually coalesced and, together with friction on the rough fracture surfaces and mechanical interlocking of micro-protrusions, triggered a second phase of strain softening, with the second peak strength approaching twice that of the first peak. In the bidirectional-grooved specimens, vertical crack propagation was predominant. The coalescence of the vertical crack produced the first peak, after which the stress continued to increase under the combined effects of friction and mechanical interlocking. It was observed that the crack shifted slightly toward the center of the specimen near the vertical midpoint. It was speculated that localized weak zones at the specimen interface might have led to rapid vertical crack propagation; in the later stages of damage, the manifestation of shear dilation caused the slope of the curve to decrease gradually. The differences in the aforementioned crack propagation and coalescence patterns, combined with the heterogeneity of the sandstone’s internal structure, collectively explain the differences in stress-strain curve shapes and strength values. Therefore, under the current test conditions, the differences in the stress-strain curve shapes and strengths among different specimens can hardly be attributed to the grooving type alone; the internal heterogeneity of the sandstone primarily influences them. The associated local weak zones and variations in crack propagation paths consequently yield different failure modes.

### 3.2. Response Characteristics of Piezoelectric Ultrasonic Signals

**Figure 7:**
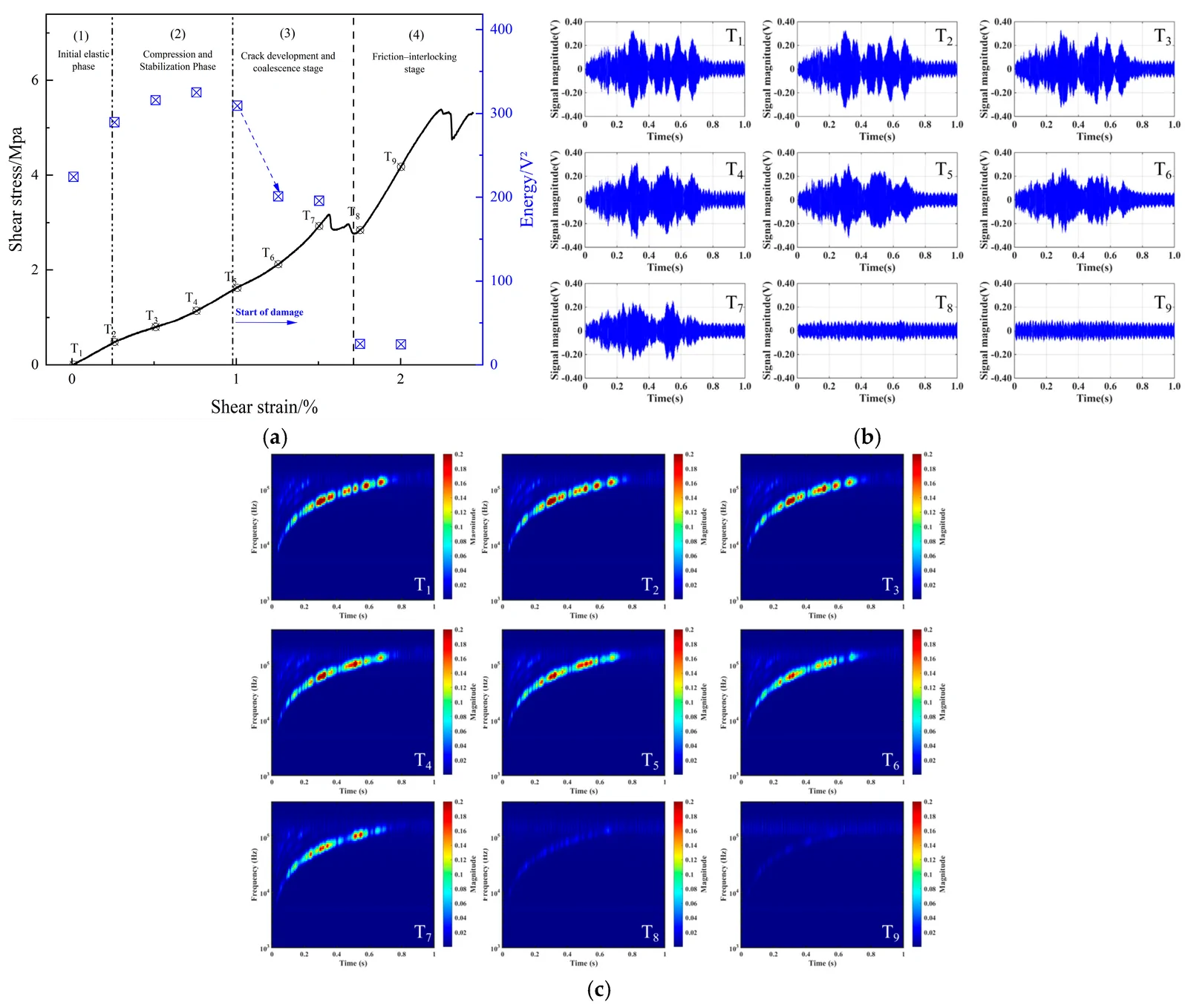
As-cut sample ultrasonic monitoring signals: (**a**) stress–strain and energy-variation curves; (**b**) time-domain signals; (**c**) CWT time–frequency signals.

Figure 7 presents the ultrasonic signals acquired during shearing of the as-cut specimen. As shown in Figure 7a, monitoring time point T_1_ was located in Stage I, T_2_–T_4_ were in Stage II, T_5_–T_7_ in Stage III, and T8 and T9 in Stage IV. As shown in Figure 7b,c, compared with T_1_ (Stage I), T_2_ (Stage II) showed essentially no change in the amplitude or waveform of the time-domain signal; however, its CWT spectrum exhibited a slight increase in signal energy in the 100–250 kHz range, indicating that after the native microcracks and pores had been compacted, ultrasonic attenuation decreased, allowing more high-frequency components to be transmitted. During Stage II, as time progressed, the amplitude of high-frequency components in the time-domain signal increased slightly, and the waveform changed accordingly, exhibiting a richer high-frequency content. The CWT spectrum revealed a further increase in signal energy in the 100–250 kHz range, indicating that the medium continued to densify, attenuation of high-frequency components continued to decrease, and the overall fidelity of the transmitted signal gradually improved. Compared with T_4_ (Stage II), the amplitude of the time-domain signal at T_5_ (Stage III) decreased, and the signal energy in the 100–250 kHz range of the CWT spectrum had noticeably weakened. These observations indicated that the structure had sustained damage; newly formed cracks increased the density of high-frequency scattering sources, causing attenuation of the transmitted high-frequency energy. During Stage III, as time progressed, the amplitude of the time-domain signal decreased further, and the energy in the 70–250 kHz band of the CWT spectrum continued to weaken. This trend reflected a continuous increase in crack density and a gradual expansion of crack size, resulting in a continuous increase in the high-frequency scattering cross-section and, consequently, more significant attenuation of the high-frequency components in the transmitted wave. At T_8_ and T_9_ (Stage IV), the time-domain signals were almost completely drowned out by noise, and their amplitudes approached the background level; the CWT spectrum exhibited energy-deficient regions across all frequency bands and time intervals, indicating that the acoustic transmission path had been essentially blocked. However, the specimen continued to bear the applied shear load through friction between the fracture surfaces and interlocking of micro-protrusions. Furthermore, the total energy in the 203.125–218.75 kHz frequency band was extracted for each time interval using the wavelet packet decomposition method described above. As shown in Figure 7a, the trend was generally consistent with the characteristics observed in the time- and frequency-domain signals, effectively reflecting the shear damage process of the bonded sandstone interface: energy increased during the compaction stage and decreased significantly upon entering the damage and failure stage.

**Figure 8:**
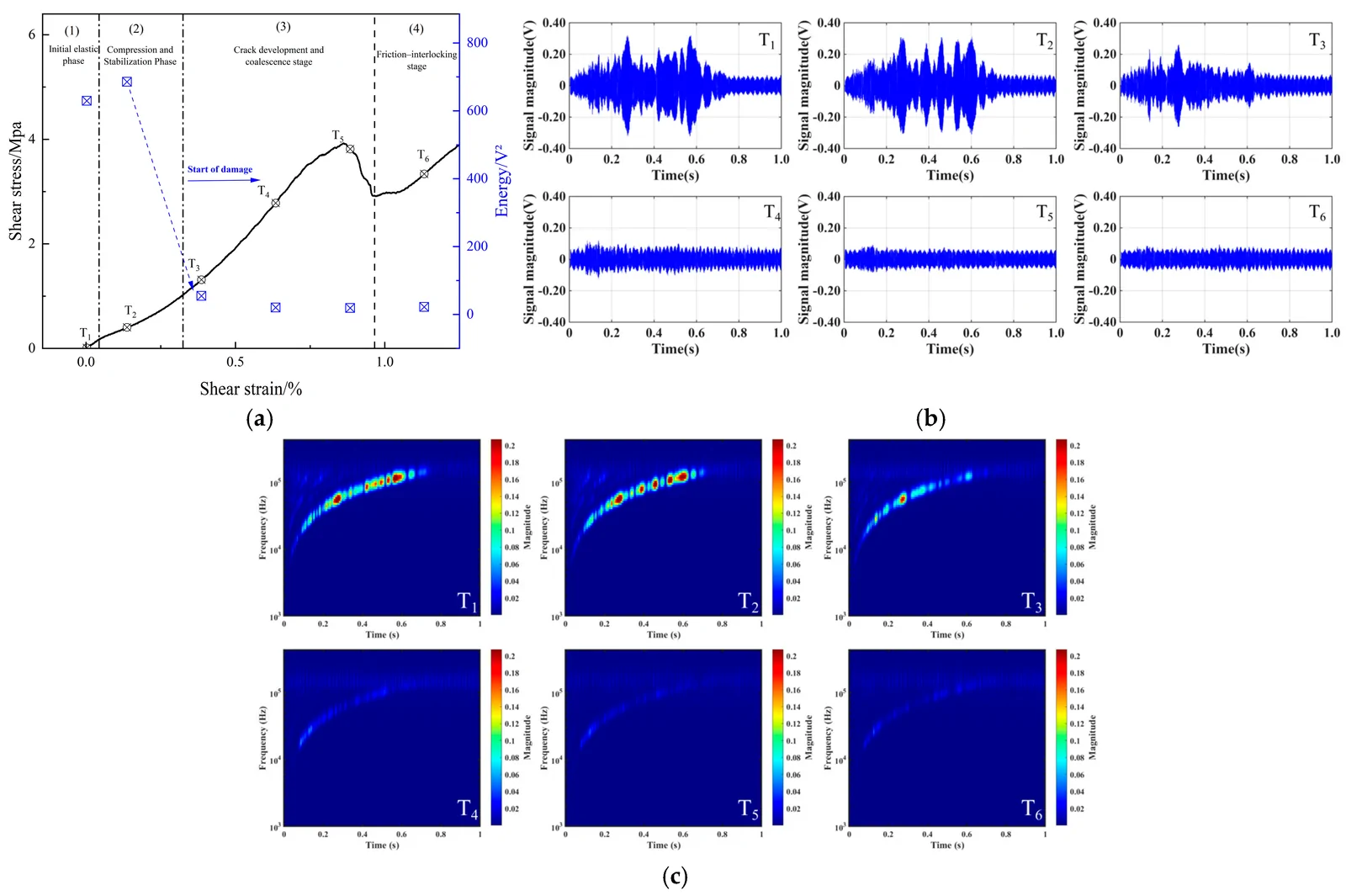
Bidirectional-grooved specimen ultrasonic monitoring signals: (**a**) stress–strain and energy-variation curves; (**b**) time-domain signals; (**c**) CWT time–frequency signals.

Figure 8 presents the ultrasonic signals acquired during shearing of the bidirectional-grooved specimen. As shown in Figure 8a, monitoring time point T_1_ was located in Stage I, T_2_ in Stage II, T_3_-T_5_ in Stage III, and T6 in Stage IV. As shown in Figure 8b,c, compared with T_1_ (Stage I), the amplitude of the time-domain signal at T_2_ (Stage II) increased, and the waveform underwent a noticeable change. The signal energy in the CWT spectrum in the 90–250 kHz range increased significantly, indicating that the specimen had entered the compaction stage and the medium was becoming denser. Upon entering Stage III, compared with T_2_ (Stage II), the amplitude of the time-domain signal in the high-frequency region at T_3_ (Stage III) had noticeably weakened, and the waveform had significantly collapsed, indicating that the structure had sustained damage. Within Stage III, as time progressed, the amplitude of the time-domain signal decreased further, and the waveform gradually collapsed to noise levels. Signal energy in the CWT spectrum in the 70–250 kHz range continued to weaken until an energy-deficient zone was observed across the entire frequency range and time period, indicating that cracks continuously formed, propagated, and eventually coalesced, completely severing the acoustic transmission path. The signal at T_6_ (Stage IV) was essentially identical to that at the end of Stage III, indicating that the acoustic transmission capability had been completely lost. Furthermore, using the method described above, the total energy in the 187.5–203.125 kHz frequency band was extracted for each time interval. As shown in Figure 8a, the trend in this energy was generally consistent with the characteristics of the time- and frequency-domain signals: the energy increased during the compaction stage. It then decreased significantly upon entering the damage and failure stage, accurately reflecting the shear damage process at the bonded sandstone interface.

In summary, although the as-cut and bidirectional-grooved specimens exhibited certain differences in shear mechanical behavior and ultrasonic signal response due to their distinct interface types, the evolution of interfacial shear damage and the characteristics of ultrasonic signal changes showed good consistency between the two specimen types. The damage evolution process drove changes in the ultrasonic signal response, while changes in the signal response could also be used to infer and characterize the damage evolution process. The above results indicated that the piezoelectric ultrasonic monitoring method could effectively monitor the damage evolution process at the bonded sandstone interface.

It is worth noting that the two frequency bands used in this study were not predefined fixed characteristic frequency bands, but rather representative characterization windows selected based on adjacent high-frequency sub-bands that were relatively sensitive to the damage evolution response in each specimen. The sensitive sub-bands corresponding to the two specimen types were not entirely identical; this is speculated to be related to differences in local contact conditions and crack propagation paths caused by variations in interface morphology. Therefore, these frequency bands are better regarded as empirical characterization windows. Furthermore, the Stage I–IV labels used in this study were neither defined retrospectively based on classification model outputs, nor were uniform, fixed stress, strain, or energy thresholds established across all specimens. Instead, a comprehensive assessment was conducted at the specimen level by integrating the mechanical response during direct shearing, ultrasonic signal characteristics, changes in wavelet packet energy, and crack evolution phenomena. In particular, the decline in load-bearing capacity following the first peak served as a key mechanical marker for the post-peak damage stage; for the pre-peak stage, representative damage evolution intervals were delineated based on the consistency of multi-source response characteristics. Owing to inter-specimen heterogeneity and local differences at the interface, the duration of each stage and the location of its boundaries were not entirely consistent among specimens.

## 4. Intelligent Identification and Monitoring of Damage Progression

### 4.1. ViT Model Establishment

**Figure 9:**
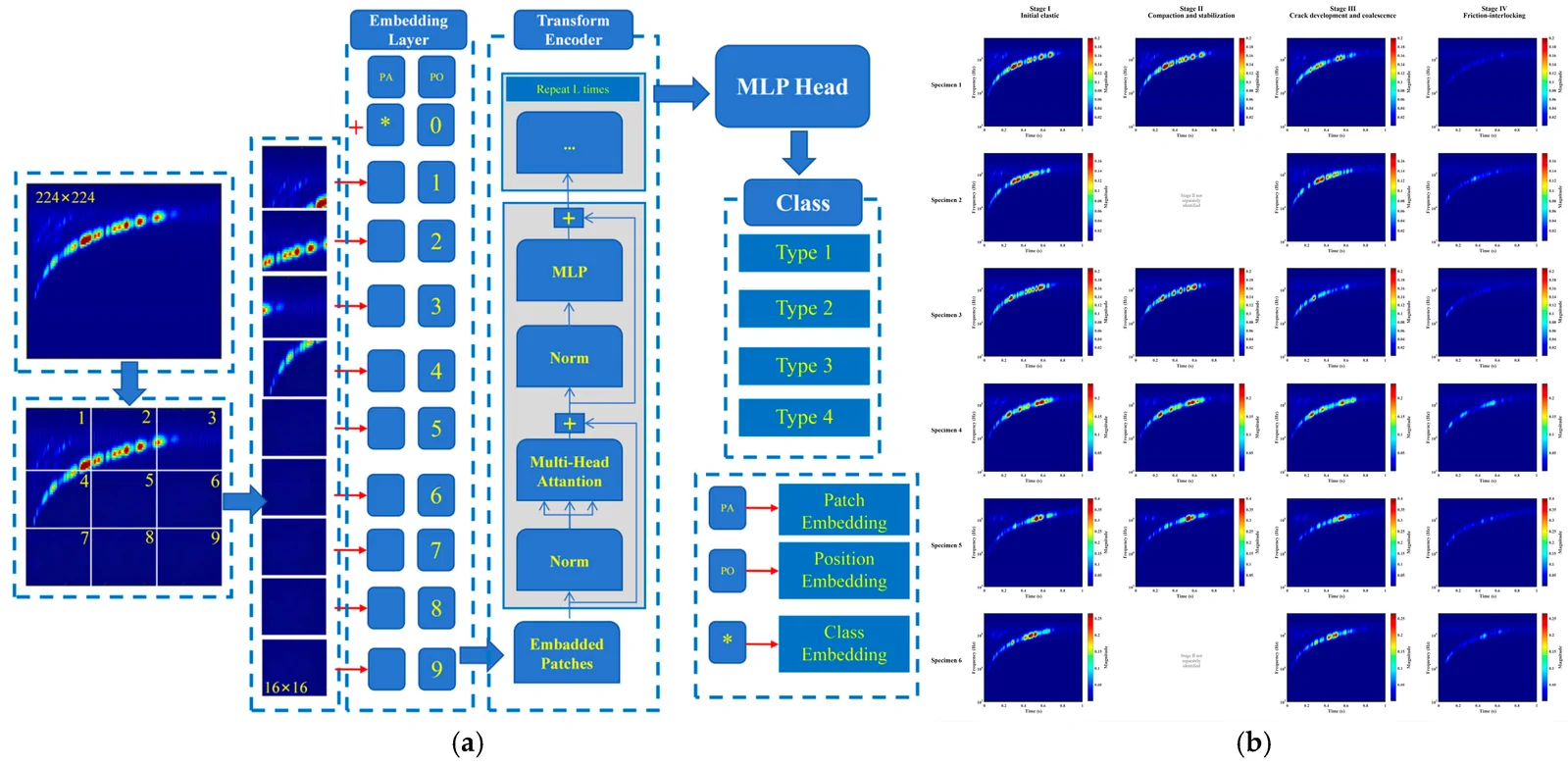
ViT deep learning model architecture and representative CWT time–frequency spectra: (**a**) ViT deep-learning model architecture; (**b**) Representative CWT time–frequency spectra of the six specimens at different damage stages. Specimens 1 and 6 are as-cut, Specimens 2 and 5 are unidirectional-grooved, and Specimens 3 and 4 are bidirectional-grooved. The panels labeled “Stage II not separately identified” indicate that the compaction–stabilization stage was not separately identified for the corresponding specimens.

ViT [63] is a landmark Transformer-based model in computer vision. Global modeling capability, a unified architecture, and excellent scalability and performance on large-scale datasets characterize it. As shown in Figure 9, its basic principle is as follows: First, the input image is divided into fixed-size patches. Each patch is flattened and linearly projected into a one-dimensional vector, which serves as the patch embedding. Simultaneously, a positional encoding is added to each patch to preserve spatial information, and a dedicated learnable classification token ([CLS] token) is introduced. Subsequently, all patch embeddings together with the classification token are fed into a stack of L standard Transformer encoder layers. Each encoder layer consists of Layer Normalization, Multi-head Self-Attention, and a Multi-Layer Perceptron (MLP). Finally, the output vector corresponding to the classification token is fed into the MLP head for classification. The basic ViT architecture and the representative CWT time-frequency spectra of the six specimens at different damage stages are shown in Figure 9.

The input image ($PI\in R^{H\times W\times C}$) is divided into *N* patches of size *P* × *P*, i.e.,:

(5) $$N=\frac{H\times W}{P\times P}$$

where H, W, and C denote the image height, width, and number of channels, respectively (for typical RGB images, C = 3).

Each image patch is flattened into a vector $PI_{p}^{i}\in R^{P\times P\times C}$ and then linearly projected to a D-dimensional vector through the Linear Projection of Flattened Patches:

(6) $$e_{i}=PI_{p}^{i}\cdot E$$

(7) $$E_{patch}=\left[e_{1};e_{2};\ldots ;e_{N-1};e_{N}\right]$$

where $E\in R^{\left(P\times P\times C\right)\times D}$ is the patch embedding projection matrix, and $E_{patch}\in R^{N\times D}$ is the resulting sequence of patch embeddings.

As in BERT, a learnable classification token ([CLS] token, $e_{CLS}$) is prepended to the sequence of patch embeddings. Learnable position embeddings $E_{pos}\in R^{\left(N+1\right)\times D}$ are then added to the patch embeddings, following the design of the standard Transformer. The resulting sequence input to the multi-head self-attention (*MSA*) is:

(8) $$X=\left[e_{CLS};e_{1};e_{2};...;e_{N-1};e_{N}\right]+E_{pos},X\in R^{\left(N+1\right)\times D}$$

The sequence is processed by a stack of L layers of multi-head self-attention (*MSA*), *MLP*, and Layer Normalization, yielding:

(9) $$X_{j}=MLP\left[Ln\left(MSA\left(Ln\left(X_{j-1}\right)\right)+X_{j-1}\right)\right]+MSA\left(Ln\left(X_{j-1}\right)\right)+X_{j-1}$$

where *Ln* represents layer normalization, MSA represents multi-head self-attention, and *MLP* represents the feedforward network.

Finally, the output vector of the [CLS] token ($e_{CLS}^{L}$) is passed through an *MLP* head with a single hidden layer to produce the classification result.

(10) $$e_{result}=MLP_{Head}\left(e_{CLS}^{L}\right)$$

Among these, multi-head self-attention is an extension of self-attention; it computes multiple attention heads in parallel on queries (*q*), keys (*k*), and values (*v*); concatenates their outputs; and then applies a linear projection. The *MLP* consists of two fully connected layers with a GELU activation function in between.

(11) $$\left[q,k,v\right]=XW_{\left(q,k,v\right)}$$

(12) $$\mathrm{SA}\left(q,k,v\right)=v\cdot \mathrm{soft}\max\left(qk^{T}/\sqrt{D/h}\right)$$

(13) $$MSA\left(q,k,v\right)=W^{0}\cdot \mathrm{Concatenation}\left(SA_{1};SA_{2};SA_{3};\ldots ;SA_{h-1};SA_{h}\right)$$

where $W_{\left(q,k,v\right)}$ is a learnable weight matrix, h is the number of heads, and $W^{0}$ is the linear projection matrix.

(14) $$\mathrm{MLP}=\mathrm{GELU}(XW_{1}+b_{1})W_{2}+b_{2}$$

where $W_{i}$ and $b_{i}$ (*i* = 1, 2) denote the weight matrix and bias vector of the *i*-th linear layer, respectively.

The experimental dataset includes multiple specimens obtained under different testing conditions. Owing to factors such as the initial state of the material, sensor coupling conditions, loading history, and signal energy scale, CWT spectra at the same damage stage may vary across specimens; meanwhile, locally similar time–frequency features may appear in adjacent or even different damage stages. These cross-specimen variations increase the difficulty of identifying interfacial damage stages under small-sample conditions. Furthermore, during the transition from Stage I to Stage II, changes in CWT spectra are typically subtle and concentrated primarily in the mid-to-high-frequency regions, making it difficult to extract these features using conventional global visual features alone reliably. Constrained by the small sample size, ViT-Base/16, with its large number of parameters, is also more prone to overfitting during fine-tuning. Preliminary experiments indicate that conventional large-parameter ViT models struggle to adequately capture the initial cross-specimen differences and the subtle frequency changes described above, which adversely affects training convergence and recognition performance on validation specimens.

To address, under small-sample conditions, the risks of overfitting, initial differences in CWT signals, and the difficulty of stably extracting subtle early-stage frequency changes in interfacial damage recognition across specimens, targeted designs were adopted in three areas: initial health reference alignment, model size control, and frequency-aware enhancement. First, to minimize initial imaging differences among specimens caused by sensor coupling conditions, signal gain, and material heterogeneity, a reference alignment strategy based on the specimen’s own Stage I health observations was employed. For each specimen, the mean and standard deviation of the health reference are calculated using its Stage I CWT spectra. The health reference from the same specimen is then used to normalize subsequent observations, thereby mitigating the impact of differences in absolute response amplitudes and enabling the model to focus more on time–frequency structural changes relative to the specimen’s own healthy state. This process uses only health observations available during the initial monitoring phase and does not use damage stage labels, mechanical responses, or future observations. For validation specimens, the Stage I observations are used only to construct the input health reference for that specimen and are excluded from model parameter updates and backpropagation. Therefore, the model evaluation in this study corresponds to a cross-specimen continuous monitoring scenario in which health references are available. Second, considering that large Transformer models are more prone to overfitting under small-sample conditions, ViT-Small/16, with a relatively moderate parameter scale, was selected as the base architecture. The CWT spectra aligned with the health reference are divided into fixed-size patches and embedded as token sequences into ViT-Small/16 to extract global visual features representing the overall time–frequency distribution and the correlations among different local regions. Third, to enhance the model’s ability to characterize subtle frequency changes and frequency-related time–frequency features, a frequency-aware module was introduced in addition to the base ViT model. This module first uses color priors from high-response regions of the CWT spectra to perform adaptive gating on patch token features, thereby adjusting the contribution of different local time-frequency regions to the subsequent representation; subsequently, the frequency axis is divided into four nominal frequency bands, and the average response, peak response, and their changes in the preceding and subsequent time intervals are calculated to construct frequency–time statistical features. After learnable projection, these features are injected into the classification representation, enabling the model to adaptively focus on different frequency regions and their temporal evolution characteristics without requiring any specific frequency band or time point to play a decisive role.

Finally, the global visual features extracted by the ViT backbone are fused with the frequency-aware features via a learnable mapping, and an MLP classification head is used to discriminate the four damage stages. The Transformer blocks, frequency-aware module, and classification head are jointly optimized with a classification loss, thereby enabling intelligent identification of the shear damage stages at bonded weak sandstone-binding material interfaces under small-sample and cross-specimen conditions.

The model adopted here is ViT-Small/16, initialized with pre-trained weights from DeiT-Small/16 trained on the ImageNet-1k dataset. The input size is 224 × 224, the patch size is 16 × 16 (196 patches, 14 × 14 grid), the embedding dimension is 384, and the model consists of 12 Transformer blocks and 6 multi-head self-attention heads, using the GELU activation function.

### 4.2. Training and Validation

The dataset used in this study consists of CWT spectra generated from ultrasonic signals acquired during valid shear tests. It is annotated according to the four stages of bonded interface damage evolution defined in Section 3. A total of 182 raw CWT spectra were used for model training and evaluation. Given the strong correlation among CWT spectra acquired at adjacent loading times for the same physical specimen, this study employs a fixed division by physical specimen prior to data augmentation: Specimens 2–5 are used for model training. In contrast, Specimens 1 and 6 are used for validation, thereby ensuring the isolation of training and validation samples. The training set and validation set contain 120 and 62 raw spectra without augmentation, respectively, and both cover all four damage stages. To avoid random division from causing adjacent observations of the same specimen or augmented versions of the same spectrum to appear across different sets, which could lead to an overly optimistic evaluation, random splitting by specimen was not applied. Given the limited number of samples and the uneven coverage of damage stages across different specimens, some validation iterations may lack specific stages, making it difficult to conduct a stable comparison of four-class classification performance. Therefore, this study adopts the aforementioned fixed-specimen validation scheme to preliminarily evaluate the model’s recognition performance on specimens not included in training under small-sample conditions, while ensuring that both the training and validation sets cover all four damage stages.

A given spectrum and its augmented versions belong exclusively to either the training set or the validation set and are not used across sets. During the training phase, 960 training samples were generated per training cycle through random sampling and mild augmentation; the light augmentation evaluation procedure set up during the validation phase generates 310 validation samples per training cycle to examine the model’s robustness to small-scale perturbations. Final performance is evaluated on raw CWT spectra without augmentation to reflect the model’s recognition performance under strict damage-stage conditions in real-world scenarios. Augmentation methods include small Gaussian noise and Gaussian blur; rotation, flipping, or extensive cropping are not used to preserve the physical meaning of the CWT time and frequency axes. The validation specimens are excluded from backpropagation and model parameter updates.

During training, the first 10 Transformer blocks are frozen, and only the subsequent layers and the newly added feature modules are fine-tuned. The AdamW optimizer is used with a base learning rate of 3 × 10^−5^, a weight decay of 0.05, and a batch size of 32. Focal Loss (γ = 1.5, label smoothing = 0.05) is adopted as the loss function, and the learning rate is dynamically adjusted based on the validation loss using the ReduceLROnPlateau strategy. All training experiments were conducted on a workstation equipped with an Intel Core i9-10900X CPU @ 3.7 GHz and an NVIDIA RTX A2000 (12 GB) GPU, using CUDA 12.6 for GPU acceleration, for a total of 50 epochs. To evaluate training randomness, training was repeated with five random seeds (42, 2024, 2025, 3407, and 7777) under the fixed specimen-wise partition, while all other configurations remained identical.

**Figure 10:**
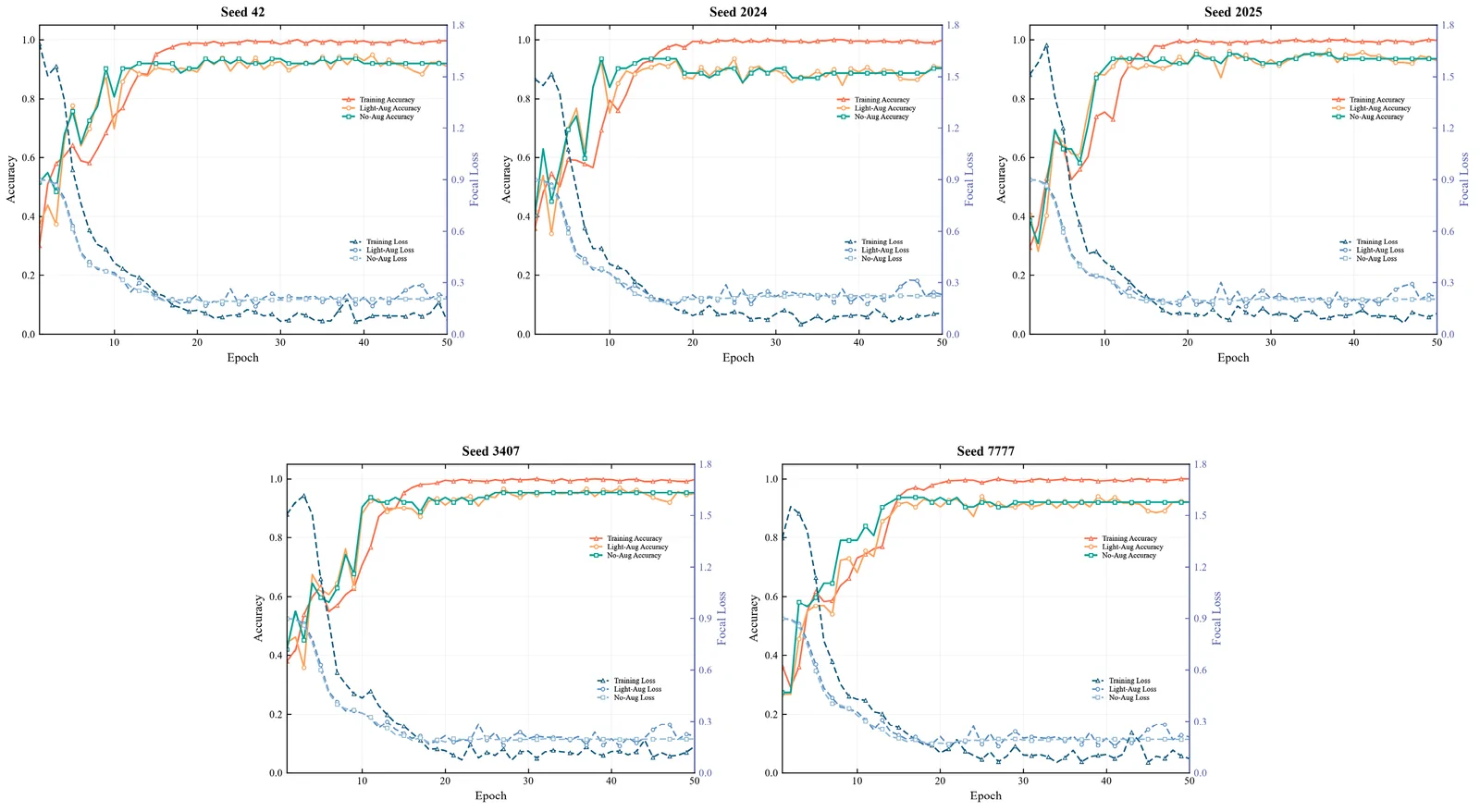
Training and validation curves across five random seeds.

Figure 10 presents the accuracy and focal loss curves from five training runs with different random seeds. The curve trends are generally consistent across the different random seeds. In each training run, the model achieved main convergence within the first 20 epochs; thereafter, the training accuracy stabilized at a high level, while the focal Loss decreased to a low level and became steady. After the main convergence phase, the performance gap between the training set and the non-augmented validation set did not continue to widen, nor was any trend of sustained decline in validation accuracy or sustained increase in focal loss observed on the non-augmented validation set. This indicates that no obvious signs of overfitting were observed under the current fixed specimen-wise validation conditions. The training curves for the non-augmented and light-augmentation validation sets were broadly similar, indicating that, within the set range of minor perturbations, the model’s stage recognition performance did not degrade significantly, demonstrating a degree of robustness to minor acquisition perturbations. The final checkpoint for each training run was selected as the epoch with the lowest validation loss on the non-augmented validation set; all selected epochs fell after the validation loss had completed its main decline, indicating that the model had reached a relatively stable convergence state. The optimal epochs across the five runs were concentrated between epochs 17 and 21, indicating a fairly consistent convergence range. Under the five random seeds, the accuracy and Loss on the non-augmented validation set were 93.23% ± 0.72% and 0.1629 ± 0.0080, respectively. The above results were obtained under a fixed specimen-wise partition, with the Stage I health observations of each target specimen serving as the health reference. Therefore, the results of this study reflect the few-shot and cross-specimen recognition performance under conditions where a health reference is available.

**Figure 11:**
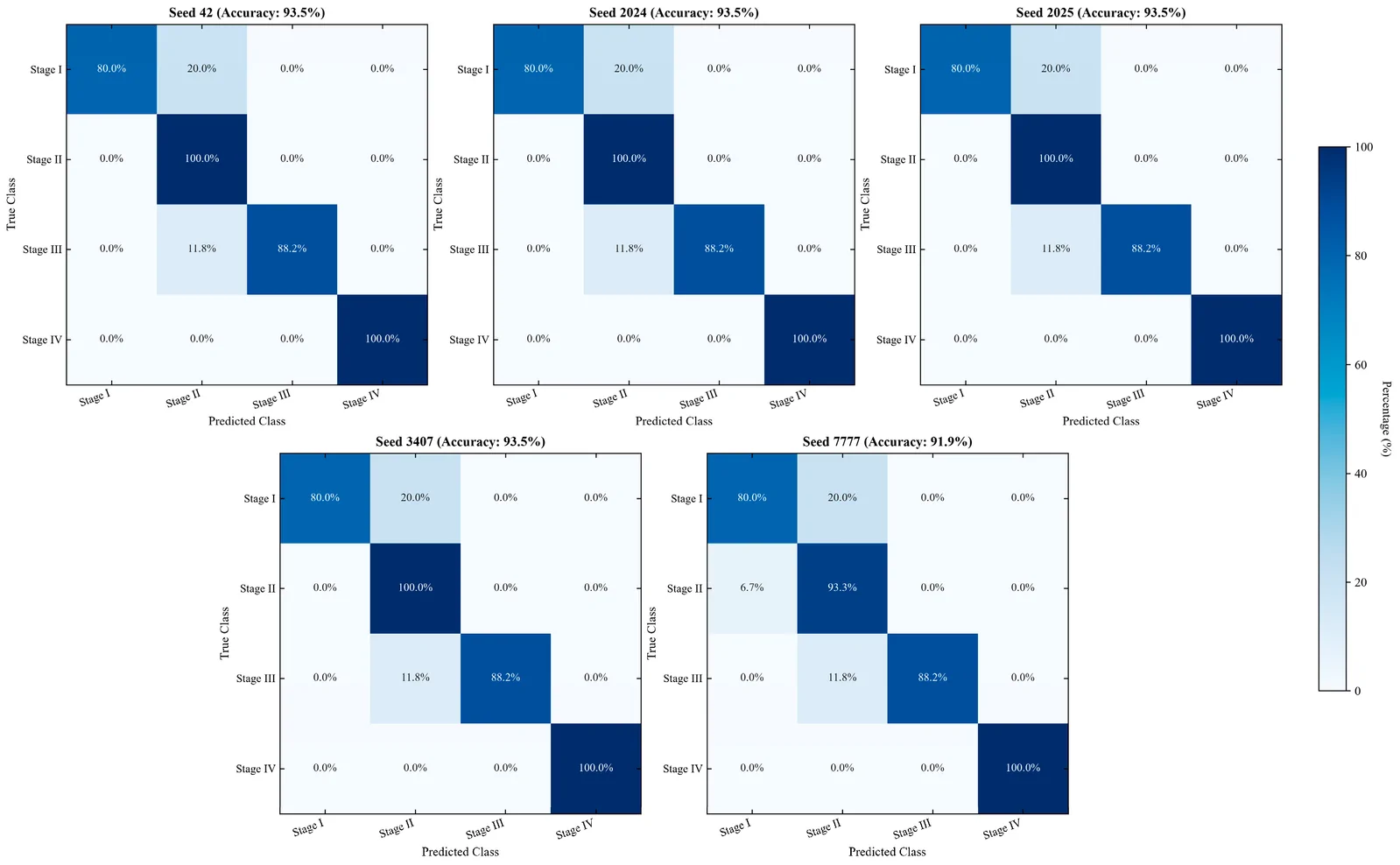
No-augmentation validation confusion matrices.

**Figure 12:**
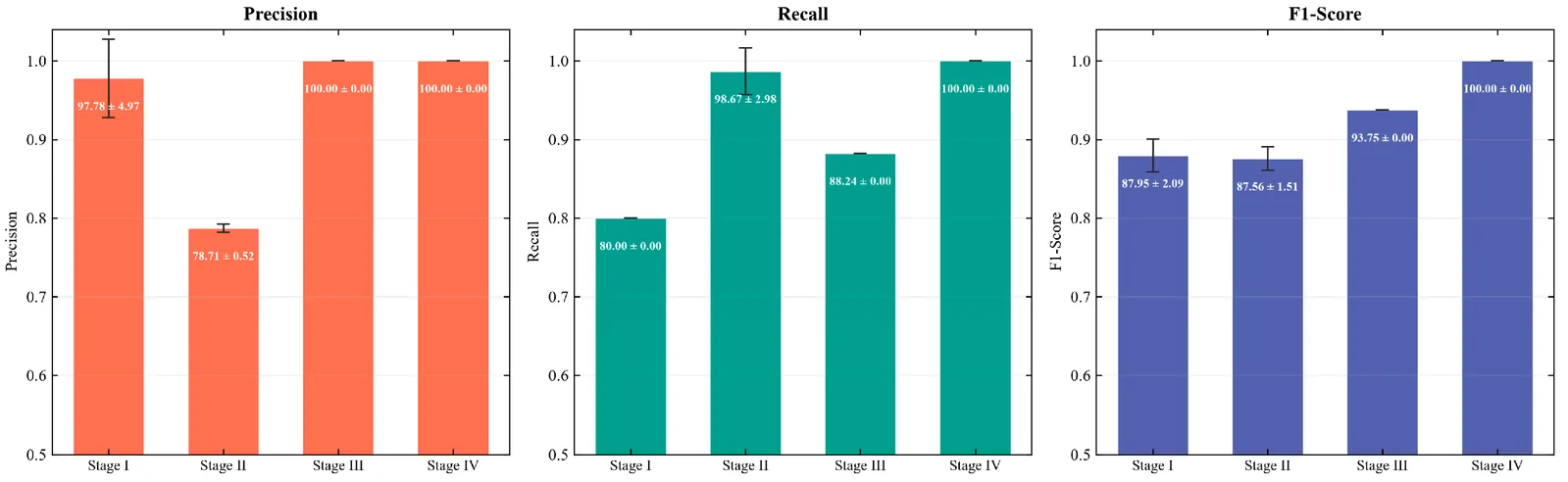
Per-stage metrics across five random seeds.

To further analyze the model’s discriminative performance across different damage stages, the confusion matrices and stage-specific metrics for the non-augmented validation set under five random seeds were compared. As shown in Figure 11 and Figure 12, the model’s errors are primarily concentrated between adjacent damage stages, with no widespread confusion spanning multiple stages. A small number of true Stage I and Stage III samples were predicted as Stage II; consequently, Stage II exhibits high recall but relatively low precision. This indicates that the model can adequately identify true Stage II samples, but it shows a certain bias toward Stage II when classifying samples near stage transitions. The observed misclassification pattern is related to the gradual evolution of interface damage. As mentioned earlier, during the transition from Stage I to Stage II, the specimen undergoes compaction and stabilization; the closure of microcracks and adjustments to the internal structure cause continuous and subtle changes in the ultrasonic response in localized time–frequency regions, rather than abrupt changes. During the evolution from Stage II to Stage III, crack initiation, local damage accumulation, and crack propagation are also gradual, and the CWT time–frequency features evolve continuously. Therefore, samples near the Stage I–II or Stage II–III transitions may simultaneously exhibit partial time-frequency features of the adjacent stages, causing Stage II to overlap to some extent with the neighboring stages in the feature space. Accordingly, the F1-scores for Stage I, Stage II, Stage III, and Stage IV are 87.95% ± 2.09%, 87.56% ± 1.51%, 93.75% ± 0.00%, and 100.00% ± 0.00%, respectively. Although the recognition performance for Stage I and Stage II remains satisfactory, their F1-scores are slightly lower than those for Stage III and Stage IV. This is primarily attributed to the relatively subtle changes in ultrasonic response during the early adjacent damage stages, which cause the two stages to lie closer to each other in the feature space.

The above results indicate that the proposed method can effectively distinguish the major stages of interface damage evolution; however, the main challenge remains identifying boundaries between adjacent stages during continuous damage processes. In future work, denser loading observations could be added in the transition regions between Stage I and II and between Stage II and III, and stage boundary discrimination could be further optimized by incorporating the continuous nature of damage evolution to reduce the classification uncertainty for boundary samples. Beyond recognition performance, practical SHM applications also require consideration of the model’s computational latency and memory consumption. This study adopts the relatively lightweight ViT-Small/16; however, because it still involves CWT preprocessing and global self-attention computations, its feasibility for deployment on resource-constrained edge devices requires further evaluation. Given that this study primarily focuses on validating recognition performance under small-sample and cross-specimen conditions, its deployment performance on specific edge hardware platforms still requires further analysis.

### 4.3. Model Performance Evaluation

To compare the proposed method with conventional hand-crafted feature-based classification methods, a machine learning pipeline based on wavelet packet energy (WPE) was developed. This pipeline employed the same training and validation partition as the proposed ViT method and adhered to the same evaluation criteria. For the raw ultrasonic signals, a three-level wavelet packet decomposition using the db4 wavelet was performed, and energy features were extracted from eight sub-bands arranged in frequency order. The eight sub-bands cover 0–500 kHz, each with a bandwidth of 62.5 kHz: 0–62.5, 62.5–125, 125–187.5, 187.5–250, 250–312.5, 312.5–375, 375–437.5, and 437.5–500 kHz. Subsequently, the energy of each sub-band was normalized by the total energy of all sub-bands to obtain an 8-dimensional WPE feature vector, which was then input into RBF-SVM, KNN, and Random Forest models, respectively. As shown in Table 1, under the same specimen-wise validation partition, RBF-SVM achieved the best performance, with an accuracy of 80.65%, a precision of 79.55%, a recall of 77.21%, and an F1-score of 76.29%. KNN and Random Forest achieved accuracies of 43.55% and 51.61%, with corresponding F1-scores of 37.56% and 36.11%, respectively. In contrast, the proposed ViT method achieved higher recognition performance across all metrics, indicating that relying on pre-extracted frequency-band energy features is insufficient to fully capture the complex time-frequency variations, which exhibit subtle local differences between different damage stages. This suggests that relying solely on pre-extracted frequency-band energy features is insufficient to fully capture the complex time-frequency variations between different damage stages. In contrast, deep feature learning from CWT spectra can adaptively extract more discriminative representations without manual feature design.

**Figure 13:**
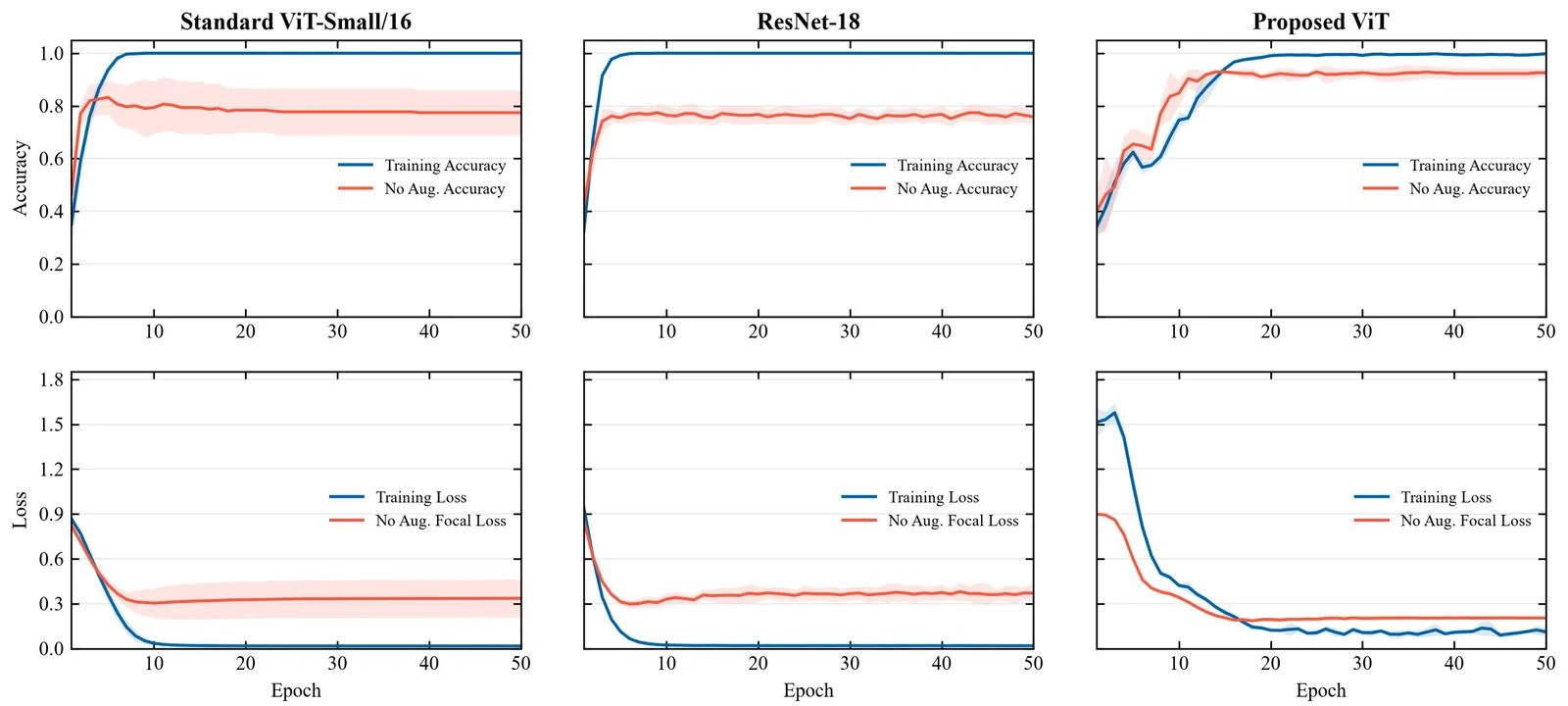
Comparison of training results for deep learning Models.

For the deep learning comparison, three models were evaluated: the standard ViT-Small/16, ResNet-18, and the proposed ViT. To ensure a fair comparison, all deep learning models followed the same dataset partition, pre-training initialization, training configuration, and evaluation protocol described above. Training was repeated with five random seeds, and the final performance was compared on the non-augmented validation set. All three models adopt the Stage I health reference strategy for each specimen. This preprocessing aims to mitigate the impact of initial state differences across specimens, enabling subsequent CWT spectra to focus more on changes relative to the specimen’s own healthy state. Moreover, the standardized preprocessing ensures that the performance differences among models primarily reflect differences in model architecture and the frequency-aware module. The standard ViT-Small/16 uses the original ViT-Small/16 architecture but does not include the frequency-aware module; ResNet-18, serving as a representative convolutional neural network reference, adopts a standard residual architecture consisting of four residual stages, each containing two basic residual blocks, and is initialized with ImageNet-1K pre-trained weights. Apart from replacing the final classification layer with a four-stage damage classification head, this model does not include a frequency-aware module; the proposed ViT combines the ViT-Small/16 architecture with the frequency-aware module. As shown in Table 1, the accuracy, precision, recall, and F1-score of the standard ViT-Small/16 were 80.32% ± 10.60%, 76.18% ± 15.00%, 79.62% ± 11.06%, and 76.91% ± 13.62%, respectively; the corresponding results for ResNet-18 were 77.42% ± 3.95%, 72.65% ± 12.41%, 75.50% ± 4.70%, and 70.22% ± 6.83%. The average performance of the two baseline models was similar, but the standard ViT-Small/16 performed slightly better overall. As shown in Figure 13, both the standard ViT-Small/16 and ResNet-18 achieved training accuracy close to 1.0 in the early stages of training, while their accuracy on the non-augmented validation set stabilized at approximately 0.77–0.80 and 0.80–0.83, respectively. Moreover, their corresponding focal loss values were relatively high and exhibited more pronounced fluctuations across different random seeds. This indicates that, given the current limited training and validation specimens, both baseline models exhibit a notable gap between training performance and validation generalization, showing a strong tendency toward overfitting. In contrast, the proposed ViT achieved accuracy, precision, recall, and F1-score of 93.23% ± 0.72%, 94.12% ± 1.37%, 91.73% ± 0.75%, and 92.32% ± 0.90%, respectively. The training curves further show that its accuracy on the non-augmented validation set stabilized at approximately 0.92, with a focal loss of 0.1629 ± 0.0080, and a narrower spread across the five random seeds. These results indicate that the combination of the ViT-Small/16 architecture and frequency-aware feature modeling contributes to improved damage stage recognition performance under small-sample conditions and enhanced stability across random seeds.

Overall, the proposed method outperforms the standard ViT-Small/16, ResNet-18, and the traditional machine learning baseline based on WPE, demonstrating a more effective use of frequency-related time–frequency information in CWT spectra. However, the results of this study reflect the cross-specimen continuous monitoring recognition performance under conditions where Stage I health reference data are available for all validation specimens. hey primarily verify the model’s preliminary applicability for cross-specimen recognition under the current small-sample setting and do not indicate universal external generalization capability in the complete absence of health reference data.

## 5. Conclusions

In this study, direct shear tests coupled with piezoelectric ultrasonic monitoring were conducted on bonded interfaces between weak sandstone and binding materials. The evolution of interface damage and its mechanical behavior were analyzed; the time-domain and time–frequency response characteristics at different damage stages were revealed, and the evolution of wavelet packet energy with damage progression was investigated. Furthermore, a method for cross-specimen interfacial damage stage identification under small-sample conditions, based on CWT time-frequency characterization and ViT, was proposed. The main conclusions are as follows:

(1) The damage evolution of weak sandstone bonded specimens under direct shear conditions can be divided into four stages: the initial elastic stage, the compaction and stabilization stage, the crack propagation and coalescence stage, and the frictional sliding and interlocking stage.
(2) The time-domain amplitude of the piezoelectric ultrasonic signal, the CWT time–frequency spectrum, and the wavelet packet energy in specific frequency bands showed good consistency with the stages of interface damage evolution. During the compaction stage, the energy of the high-frequency components increased; upon entering the damage and failure stages, the signal attenuated significantly, and energy-deficient regions appeared in the CWT time–frequency spectrum. For the as-cut and bidirectional-grooved specimens, the changes in wavelet packet energy in the 203.125–218.75 kHz and 187.5–203.125 kHz frequency bands, respectively, correlated well with the damage progression, indicating that the piezoelectric ultrasonic method can effectively monitor damage evolution at the weak sandstone–binding material interface.
(3) Based on the ViT-Small/16 architecture, combining Stage I health reference alignment and a frequency-aware module, a CWT-ViT model was developed for interfacial damage stage identification. Under fixed specimen-wise validation conditions, the proposed ViT achieved an accuracy of 93.23% ± 0.72% and a macro F1-score of 92.32% ± 0.90%. Results from five repeated runs with different random seeds demonstrate that the model exhibits favorable recognition performance and stability under the current small-sample and cross-specimen conditions.

The above results validate the feasibility of combining piezoelectric ultrasonic monitoring with deep learning models for identifying damage stages at binary interfaces. The model can extract features related to interface damage evolution from CWT time–frequency spectra, providing an intelligent technical approach for active structural health monitoring of bonded interfaces between weak sandstone and binding materials. However, the current work still has certain limitations. The experiments considered only epoxy adhesive and static direct shear conditions, with limited coverage of interface roughness types, material parameters, and loading conditions. Due to the limited sample size and the uneven coverage of different damage stages, a fixed specimen partition was adopted, and repeated training was performed with five random seeds under this partition to characterize the performance fluctuations caused by random initialization, training sampling, and random augmentation under the current small-sample conditions. The results presented in this study reflect only the cross-specimen continuous monitoring and recognition performance of the model under a fixed-specimen validation setup, with the Stage I health reference available during the initial monitoring phase of each validation specimen. This does not equate to universal external generalization capability under conditions where health references are completely absent. Future work will further evaluate and optimize the cross-specimen recognition performance of the model by adding independent physical specimens and labeled observations, expanding the range of interface parameters and loading conditions, and conducting denser observations in stage transition regions. Furthermore, the results presented in this study were obtained under controlled laboratory conditions; the impact of actual field noise, moisture conditions, and other environmental variations on model robustness has not yet been systematically evaluated. Therefore, its applicability to complex field conditions requires further validation in future work.

## Figures and Tables

**Table 1:** Performance comparison of different methods on the non-augmented validation set.

Method		Accuracy/%	Precision/%	Recall/%	F1-Score/%
Traditional Machine Learning	RBF-SVM	80.65	79.55	77.21	76.29
	KNN	43.55	42.35	37.35	37.56
	Random Forest	51.61	33.11	42.65	36.11
Deep Learning	Standard ViT-Small/16	80.32 ± 10.62	76.18 ± 15.15	79.61 ± 10.66	76.91 ± 13.69
	ResNet-18	77.42 ± 3.95	72.65 ± 12.41	75.50 ± 4.70	70.22 ± 6.83
	Proposed ViT	93.23 ± 0.72	94.12 ± 1.37	91.73 ± 0.75	92.32 ± 0.90

## Data Availability

All core data and original contributions presented in this study are directly included in the article (including figures, tables, and main text). No additional data have been made publicly available in external repositories. For further inquiries or more detailed information, please contact the corresponding author.
